# Artificial Neural Network Based Non-linear Transformation of High-Frequency Returns for Volatility Forecasting

**DOI:** 10.3389/frai.2021.787534

**Published:** 2022-02-11

**Authors:** Christian Mücher

**Affiliations:** ^1^Chair of Statistics and Econometrics, University of Freiburg, Freiburg, Germany; ^2^Graduate School of Decision Sciences, University of Konstanz, Konstanz, Germany

**Keywords:** neural networks, forecasting, high-frequency data, realized volatility, mixed data sampling, long short term memory

## Abstract

This paper uses Long Short Term Memory Recurrent Neural Networks to extract information from the intraday high-frequency returns to forecast daily volatility. Applied to the IBM stock, we find significant improvements in the forecasting performance of models that use this extracted information compared to the forecasts of models that omit the extracted information and some of the most popular alternative models. Furthermore, we find that extracting the information through Long Short Term Memory Recurrent Neural Networks is superior to two Mixed Data Sampling alternatives.

## 1. Introduction

The volatility, the time-varying centered second moment of a financial asset, is crucial to measure, forecast, and evaluate financial risk. There exist many ways of modeling and forecasting volatility separable into two main groups: Models based on (squared) daily returns and models based on the Realized Volatility (RV) estimator. In the first group, volatility is treated as a latent variable and estimated from the model. Famous examples in this regard are, on the one hand, (G)ARCH models (Engle, 1982; Bollerslev, 1986) and their various extensions that treat volatility as conditionally observable. On the other hand, Stochastic Volatility models (Taylor, 1986; Ruiz, 1994) treat conditional volatility as random variables and rely on filtering techniques for estimation and forecasting. While the vast models in this group capture stylized properties of financial data such as volatility clustering, long-memory, and asymmetric reactions of volatility to positive and negative shocks, they generally perform worse in forecasting volatility compared to the models of the second group (Andersen et al., 2004; Sizova, 2011).

The availability of high-frequency (HF), intraday returns and the introduction of RV as an estimator of integrated volatility over a day (Andersen et al., 2001a,b; Barndorff-Nielsen and Shephard, 2002a,b) lead to the second group of models. Since the RV ex-post gives a consistent volatility estimate, the main focus of models in the second group is forecasting. Andersen et al. (2001a,b), and Barndorff-Nielsen and Shephard (2002a) find that RV and the logarithm of RV exhibit long memory. Their autocorrelation functions show a hyperbolic decay, meaning that past shocks have a very long influence on the system of RV. Therefore, the authors propose forecasting volatility via fractional integrated autoregressive moving average (ARFIMA) models to account for the long memory. The most prominent alternative to ARFIMA models for forecasting volatility based on RV is the Heterogeneous Autoregressive Model (HAR) by Corsi (2009). The HAR model approximates the long memory in the data through RV's daily, weekly, and monthly averages. These averages are used in a linear model as explanatory variables to predict volatility. Corsi (2009) finds that the HAR model performs better than the ARFIMA models in forecasting volatility. The HAR model is popular because of its good performance and ease of implementation (the HAR can be estimated by simple OLS regression). There exist many extensions of the HAR model in the literature, such as the HAR with jumps model of Andersen et al. (2007), the Semivariance HAR of Patton and Sheppard (2015), or the HARQ of Bollerslev et al. (2016). However, the standard HAR model, both for the level and the logarithm of RV, still is a challenging benchmark to beat in applications on real financial data.

Artificial Neural Networks (ANNs) have become more and more popular over the last decade, and various fields apply them for classification, prediction, and modeling tasks. Cybenko (1989) and Hornik et al. (1989) show the capability of Feed Forward Neural Networks (FNNs), fully connected ANNs with one hidden layer, to approximate any continuous function on a compact set arbitrarily well. Furthermore, Schäfer and Zimmermann (2006) show that Recurrent Neural Networks (RNNs) can approximate any open, dynamic system arbitrarily well. The popularity of ANNs is, on the one hand, due to these theoretical results. On the other hand, ANNs have been among the winning algorithms for various classification and forecasting competitions over the past years. RNNs combine the ability of ANNs to capture complex non-linear dependencies in the data with capturing temporal relationships. Long Short Term Memory (LSTM) RNNs (Hochreiter and Schmidhuber, 1997) are a type of RNN specifically designed to capture long memory in data. Their capacity to capture non-linear, long-term dependencies in the data make them the perfect candidates for modeling volatility.

This paper aims to use LSTMs to non-linearly transform the HF returns of a financial asset, observed within a day, into a daily, scalar variable and to use this variable to forecast volatility. Non-linear transformations of the HF returns are not novel since the RV estimator (the sum of the squared HF returns of a day) is also a non-linear transformation, but a particular one. We investigate whether volatility forecasts solely constructed from the ANN-based transformation of the HF returns are different from forecasts obtained through the past RVs. While the ANN transformation is very flexible in the functional form, the resulting sequence might not capture the long persistence in the volatility, as the RV estimator does. However, the flexibility of the functional form might capture other information that is useful to predict volatility and that the RV estimator does not take into account. Examples of such information are the sign of the HF returns or patterns of HF returns occurring over a day. We thus combine the two approaches and investigate whether the resulting model exhibits a superior forecasting performance compared to the models that rely on each measure alone.

An alternative approach to transforming the HF returns is the Mixed Data Sampling (MIDAS) approach of Ghysels et al. (2004). In MIDAS, the transformation happens through a weighted sum of the HF returns. The weights are obtained non-linearly, e.g., by an Almon or a Beta Lag Polynomial (Ghysels et al., 2004). We introduce a novel type of MIDAS model that obtains those weights through an LSTM cell. In MIDAS applications, however, the construction of the transformed HF measure is linear.

Though, as mentioned earlier, the RV estimator is also a transformation of the HF returns, throughout the paper, we will use the term transformed HF returns or transformed measure to refer to the scalar variable obtained through either the ANN transformation or the MIDAS transformation.

We compare the forecasting performance of models that use either one of the transformed measures to forecast volatility with each other and with models that construct the forecasts relying solely on information from past RV, such as the HAR model. We can thus answer whether the transformation can extract at least the same information as the past RV. We further compare these models' forecasts with those obtained from models that combine the RV information with the transformed measures, allowing us to investigate whether the transformed measures contain information supplementary to the RV. Lastly, we can compare the different transformation methods to determine whether the non-linearity introduced through ANNs performs differently from the MIDAS approaches.

The remainder of this paper is structured as follows: section 2 gives an overview of the literature in volatility forecasting with ANNs. Section 3 introduces the LSTM RNN, and section 4 explains the different transformations of the HF returns. It first describes the non-linear transformation through LSTMs and then shows the two MIDAS approaches. Section 5 elaborates on using the transformed HF returns to generate volatility forecasts. We further introduce the benchmark models to which we compare our proposed methodology. Finally, we present the results of our empirical application in section 6, and section 7 concludes.

## 2. Literature Review

A vast area of finance applies ANNs. For example, White (1988), among others, uses ANNs to predict stock returns while Gu et al. (2020) use ANNs for asset pricing and Sadhwani et al. (2021) apply ANNs for mortgage risk evaluation. ANNs are further applied to model and forecast financial risk. The literature in this field reflects the two main branches of volatility modeling and forecasting mentioned in section 1: models based on daily (squared) returns and models based on realized measures estimated from the HF returns. An early contribution to the literature of volatility modeling and forecasting through daily squared returns is Donaldson and Kamstra (1997). The authors introduce a semi nonparametric non-linear GARCH model based on ANNs and show superior performance to other GARCH type alternatives. Franke and Diagne (2006) show that ANNs yield non-parametric estimators of the conditional variance function of an asset when trained with daily returns as inputs and squared returns as targets. Their results have been applied by Giordano et al. (2012) and generalized for the Multi-Layer-Perceptron (MLP), fully connected ANNs with multiple hidden layers, by Franke et al. (2019). Arnerić et al. (2014) exploit the non-linear Autoregressive Moving Average (ARMA) structure of a Jordan type RNN (Jordan, 1997) and the ARMA representation of the GARCH model to introduce the Jordan GARCH(1,1) model. Their model shows superior performance in out-of-sample root mean squared error (RMSE). Alternative approaches use the output of GARCH models, potentially combined with other explanatory variables, as inputs to an MLP (see e.g., Hajizadeh et al., 2012; Kristjanpoller et al., 2014).

The literature on forecasting volatility via ANNs through realized measures consists of two main fields. The first field uses ANNs to relax the linearity of the HAR model by feeding the lagged daily, weekly, and monthly averages of RV to MLPs. The evidence in this branch is mixed. Rosa et al. (2014) find improvements in the forecasting performance of the non-linear HAR model, while Vortelinos (2017) concludes that the ANN HAR model is not predicting volatility better than the linear HAR. He argues that the MLP cannot capture the long-term dependencies in the RV. Baruník and Křehlík (2016) find mixed evidence of the ANN HAR model for the volatility of energy market prices. Their ANN-based model produces more accurate forecasts than the linear model for some forecasting horizons and some markets. Arnerić et al. (2018) find that an MLP fitted to the HAR inputs can outperform the linear benchmark. In addition, they find that including jump measures in the analysis further improves the forecasting performance. Christensen et al. (2021) find superior forecasting performance of their MLP HAR model over the linear HAR. Further, they find that the model's performance improves when additional firm-specific and macroeconomic indicators are added. Li and Tang (2021) apply an MLP to a large set of variables such as realized and MIDAS measures and option Implied Variances. They find that the resulting model outperforms the linear benchmark. The performance improves further through an ensemble learning algorithm that combines the outputs of other linear and non-linear machine learning techniques, such as penalized regression and random forests, with the output from the ANN model.

The second field in the literature utilizes RNNs to capture, in addition to non-linearity, long-term dependencies in the data. Miura et al. (2019) examine the volatility of cryptocurrencies finding that a ridge regression yields the best out of sample forecasting results, followed by LSTM RNNs. Baştürk et al. (2021) apply LSTM RNNs to the past measure of RV and the negative part of past daily returns to jointly forecast the volatility and the Value at Risk (VaR) of a financial asset. The authors find superior forecasting performance of the LSTM network for the VaR forecasts. However, their approach cannot produce improved volatility forecasts compared to the linear alternatives.

A recent contribution to both branches of this literature is Bucci (2020), who compares the forecasting performance of various ANN structures to standard benchmarks from the financial econometrics literature such as the HAR model and ARFIMA models. He further investigates how adding macroeconomic and financial indicators as exogenous explanatory variables improves the model's forecasting performance. The target variable in his analysis is the monthly log square root of the RV. He finds that the long memory type ANNs such as the LSTM network outperform the financial econometrics literature's classical models. Furthermore, these models outperform the ANNs that do not account for long memory in the data. This result holds for various forecasting horizons.

Finally, Rahimikia and Poon (2020a) and Rahimikia and Poon (2020b) propose a HAR model augmented by an ANN applied to HF limited order book information and news sentiment data. In both papers, the authors find a superior forecasting performance of their model compared to the HAR benchmark. Their approach of augmenting the HAR model by transformed HF data is similar to the idea of this paper. In parts of our application, we augment models for LF measures such as the HAR with transformed HF information. The difference is that we consider the HF returns and not other auxiliary HF information. Further, we also consider models that use only the information from the transformed HF returns for the forecast.

## 3. Long Short Term Memory

LSTM RNNs are a specific type of RNN structures that overcome the problem that classical RNNs face. Specifically, the limited capacity of such networks to learn long-term relationships due to vanishing or exponentially increasing gradients (Hochreiter, 1991; Bengio et al., 1994). The cornerstone of LSTMs is the long memory cell denoted by *C*_τ_. A candidate value of which, ${\tilde{C}}_{\tau }$ is a non-linear transformation (using the hyperbolic tangent activation function *tanh*^1^) of a linear combination of the τ-th periods' input vector values *v*_τ_ and the previous periods' output value *y*_τ−1_ plus an intercept


(1) $$\begin{array}{l} {\tilde{C}}_{\tau }=tanh\left(\Theta_{C}\left[y_{\tau -1},v_{\tau }\right]+c_{C}\right). \end{array}$$

Next, the values of the forget *f*_τ_ and the input *i*_τ_ gate are computed. These are obtained by applying the sigmoid activation function σ(·)^2^ to a linear combination of the input vector values *v*_τ_ and the previous periods' output value *y*_τ−1_ plus an intercept.


(2) $$\begin{array}{l} f_{\tau }=\sigma \left(\Theta_{f}\left[y_{\tau -1},v_{\tau }\right]+c_{f}\right) \end{array}$$

(3) $$\begin{array}{l} i_{\tau }=\sigma \left(\Theta_{i}\left[y_{\tau -1},v_{\tau }\right]+c_{i}\right) \end{array}$$

The memory cell value is computed by


(5) $$\begin{array}{l} C_{\tau }=f_{\tau }C_{\tau -1}+i_{\tau }{\tilde{C}}_{\tau }, \end{array}$$

i.e., it combines the previous periods' cell value and the current periods' candidate cell value. Since the sigmoid function returns values on the interval (0, 1), *f*_τ_ denotes the share to be “forgotten” from the previous cell state and *i*_τ_ the share of the proposal state to be added to the new cell state. The output of the LSTM cell *y*_τ_ is generated by applying the *tanh* function to the memory cell values and multiplying the result by the value of the output gate *o*_τ_


(6) $$\begin{array}{l} y_{\tau }=o_{\tau }\psi \left(C_{\tau }\right), \end{array}$$

where the latter is obtained in the same manner as the values of *f*_τ_ and *i*_τ_


(7) $$\begin{array}{l} o_{\tau }=\sigma \left(\Theta_{o}\left[y_{\tau -1},v_{\tau }\right]+c_{o}\right). \end{array}$$

The output gate gives the share of the activated cell values to return as the output of the memory cell. LSTM cells thus are dynamic systems wherein interacting layers drive the hidden state dynamics. This interaction enables the LSTM cell to account for a high degree of non-linearity and to capture long-term dependencies in the data.

## 4. Transformation of the High-Frequency Returns

Denote by *r*_*t,j*_ the *t*-th days' *j*-th log-return. We have *j* = 1, …, *M* equidistantly sampled returns within day *t*. The increments between two intraday returns determine the number of intraday observations. For returns sampled every 5 minutes within a normal trading day at the New York Stock Exchange, we obtain 78 intraday high-frequency returns. We will denote the vector of the intraday returns on day *t* by *r*_*t*,1 : *M*_. We aim to apply a transformation to *r*_*t*,1 : *M*_ that returns a scalar value ${\tilde{x}}_{t}\left(\theta^{HF}\right)$ which we will refer to as the transformed measure. The transformation depends on parameter vector θ^*HF*^. We present three different methods to obtain the transformed measure in the following.

### 4.1. Non-linear Transformation

The LSTM architecture described earlier can be used for a non-linear transformation of the HF returns. In its' simplest form, we use the sequence of the *M* intraday returns as input to the LSTM cell, and the output of the cell at time *M*, *y*_*t,M*_, as the transformed value. We thus iterate through the LSTM equations over the *j* = 1, …, *M* intraday returns at day *t*


(8) $$\begin{array}{l} {\tilde{C}}_{t,j}=tanh\left(\Theta_{C}\left[y_{t,j-1},r_{t,j}\right]+c_{C}\right) \end{array}$$

(9) $$\begin{array}{l} f_{t,j}=\sigma \left(\Theta_{f}\left[y_{t,j-1},r_{t,j}\right]+c_{f}\right) \end{array}$$

(10) $$\begin{array}{l} i_{t,j}=\sigma \left(\Theta_{i}\left[y_{t,j-1},r_{t,j}\right]+c_{i}\right) \end{array}$$

(11) $$\begin{array}{l} C_{t,j}=f_{t,j}C_{t,j-1}+i_{t,j}{\tilde{C}}_{t,j} \end{array}$$

(12) $$\begin{array}{l} o_{t,j}=\sigma \left(\Theta_{o}\left[y_{t,j-1},r_{t,j}\right]+c_{o}\right) \end{array}$$

(13) $$\begin{array}{l} y_{t,j}=o_{t,j}\psi \left(C_{t,j}\right), \end{array}$$

and set ${\tilde{x}}_{t}\left(\theta^{HF}\right)=y_{t,M}$, where θ^*HF*^ contains the LSTM cell weights and intercepts. Through the interaction of the three gates and the non-linear activation of the proposal state and the actual state, the LSTM cell allows for a high degree of non-linearity while also capturing long memory in the data. Both the cell input (*r*_*t,j*_) and output (*y*_*t,j*_) at within day lag *j* are scalars. The parameter vector θ^*HF*^ of the model using one LSTM cell thus contains 12 parameters: Four 2 × 1 weight vectors and four intercepts. To increase the degree of non-linearity, we further use a network that consists of one hidden layer of LSTM cells and use the outputs of these cells as inputs to another LSTM cell returning a scalar value.

### 4.2. MIDAS Transformations

Denote by $\omega_{j}\left(\theta^{HF}\right)$ the weights associated with the *j*-th intraday return on day *t*. The weights are determined by the elements of θ^*HF*^. While the weights may be obtained in a non-linear manner, the resulting transformed measure


(14) $$\begin{array}{l} {\tilde{x}}_{t}\left(\theta^{HF}\right)=\overset{M}{\underset{j=1}{\sum }}w_{j}\left(\theta^{HF}\right)r_{t,j} \end{array}$$

is a weighted sum and thus linear.

In a Beta Lag MIDAS model (labeled Beta MIDAS hereafter), the weight associated with the *j*-th lag is obtained by


(15) $$\begin{array}{l} \omega_{j}\left(\varphi_{1},\varphi_{2}\right)=\frac{B\left(\frac{j}{M},\varphi_{1},\varphi_{2}\right)}{\overset{M}{\underset{j=1}{\sum }}B\left(\frac{j}{M},\varphi_{1},\varphi_{2}\right)} \end{array}$$

where $B\left(\cdot \frac{j}{M},\varphi_{1},\varphi_{2}\right)$ is the probability density function (pdf) of the Beta distribution. In this case, the parameter vector $\theta^{HF}={\left(\varphi_{1},\varphi_{2}\right)}^{\prime }$ contains the Beta distribution parameters. While only depending on two parameters, the weights obtained from the normalized Beta pdf are capable to capture complex non-linear functional forms.

An alternative way to obtain the weights associated with the *j*-th observation is to use the lag values as inputs to an LSTM cell. In this case, the input to the LSTM cell is *r*_*j*_ = *j* with *j* = 1, 2, …, *M*. The corresponding output (*y*_*j*_) lies on the interval between (−1, 1). Note that in this case, *r*_*j*_ and *y*_*j*_ do not depend on *t* since they only vary within the day but not over the days. To transform the output at within day lag *j* (*y*_*j*_) into a weight, we apply the *exponential* function and normalize the values, i.e.,


(16) $$\begin{array}{l} w_{j}=\frac{\exp\left(y_{j}\right)}{\overset{M}{\underset{j=1}{\sum }}\exp\left(y_{j}\right)}, \end{array}$$

yielding weights that lie in the interval (0, 1) and sum up to one. This transformation is similar to the Beta MIDAS, where the Beta pdf values associated with *j*/*M* are normalized such that they sum up to one. Same as above, the parameter vector θ^*HF*^ of the LSTM MIDAS model contains 12 parameters.

## 5. Volatility Forecasting

This paper aims to forecast the daily volatility of a financial asset. Consider the price process of a financial asset *P*_*t*_, determined by the stochastic differential equation


(17) $$\begin{array}{l} d\ln\left(P_{t}\right)=\mu_{t}dt+\sigma_{t}dW_{t} \end{array}$$

where μ_*t*_ and σ_*t*_ denote the drift and the instantaneous or spot volatility process, respectively, and *W*_*t*_ is a standard Brownian motion. The integrated variance from day *t* − 1 to *t* is then defined as


(18) $$\begin{array}{l} IV_{t}=\overset{t}{\underset{t-1}{\int }}\sigma_{s}^{2}ds. \end{array}$$

The integrated variance yields a direct measure of the discrete time return volatility (Andersen et al., 2004), but the series is latent, and we can not observe it directly. However, we can estimate the integrated variance ex-post through the RV estimator defined as


(19) $$\begin{array}{l} RV_{t}=\overset{M}{\underset{j=1}{\sum }}r_{t,j}^{2}, \end{array}$$

i.e., the sum of the *M* squared intraday HF returns. Our goal is to assess how the information obtained from applying the different transformations of the HF returns explained earlier helps predict one step ahead volatility. To answer this, we consider different scenarios.

**Figure 1:**
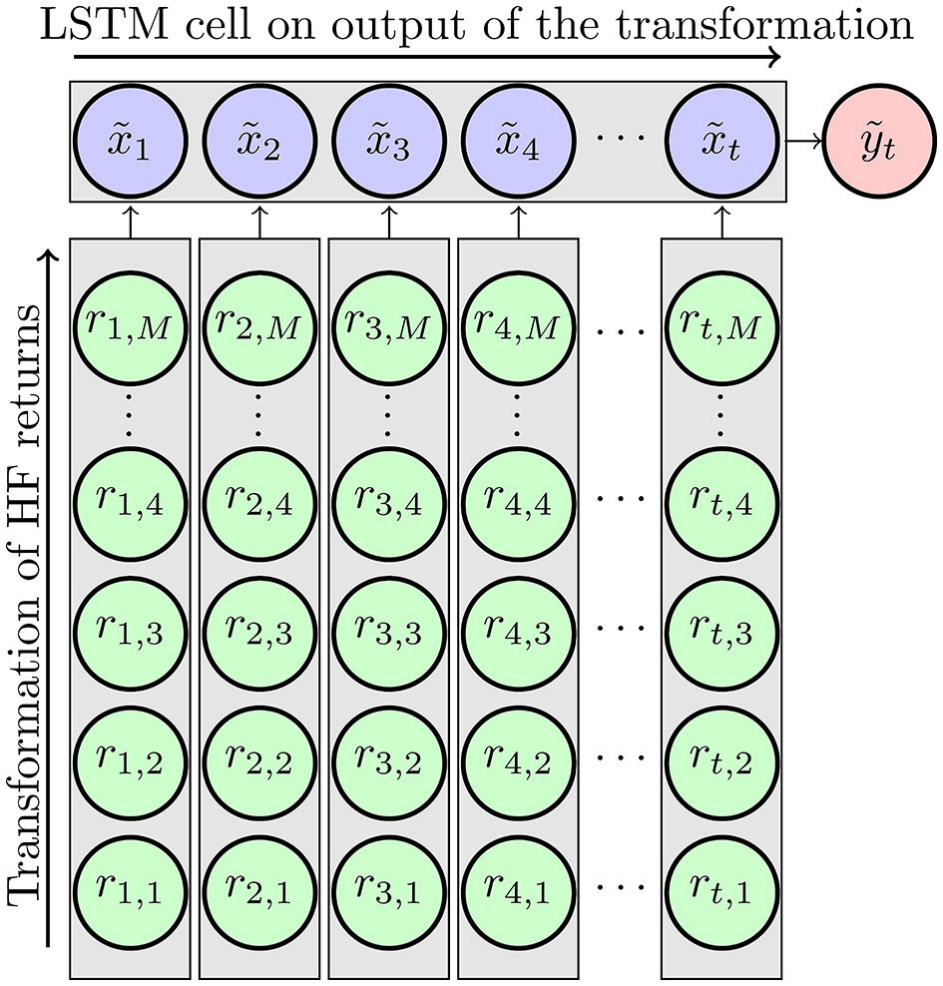
Method depicted. The HF returns are transformed in the vertical direction, meaning that for each day 1, …, *t* the same type of transformation is applied to the HF returns of that day. The result is a sequence of τ = 1, …, *t* transformed measures ${\tilde{x}}_{\tau }$ to which an LSTM cell is horizontally applied.

First, we vary the input variables used to predict volatility, considering three different settings. We start by combining the transformed measure ${\tilde{x}}_{t}$ (for readability, we omit the dependence of the transformed measure on θ^*HF*^ from here on) with past information on the RV. Next, we assess how this combination fares compared to using each stream of information on itself, i.e., using only the information obtained from the transformation and using only the past information on RV. Finally, when using the information on the transformed measure, we again differentiate between two settings: In the first, we only use the most recent (the past days) value of the transformed measure. In the second, we account for dynamics in the transformed measure and use the values of multiple past days. The contributions of Andersen et al. (2001a) and Andersen et al. (2001b) as well as models like the HAR and the work by, e.g., Audrino and Knaus (2016), show that it is necessary to account for the long memory in the volatility. In the setting where we use multiple past values of the transformed measure, we therefore apply an LSTM cell to the sequence of transformed measures. This means that for τ = 1, …, *t*, we iterate over


(20) $$\begin{array}{l} {\tilde{C}}_{\tau }=tanh\left(\Theta_{C}\left[y_{\tau -1},{\tilde{x}}_{\tau }\right]+c_{C}\right) \end{array}$$

(21) $$\begin{array}{l} f_{\tau }=\sigma \left(\Theta_{f}\left[y_{\tau -1},{\tilde{x}}_{\tau }\right]+c_{f}\right) \end{array}$$

(22) $$\begin{array}{l} i_{\tau }=\sigma \left(\Theta_{i}\left[y_{\tau -1},{\tilde{x}}_{\tau }\right]+c_{i}\right) \end{array}$$

(23) $$\begin{array}{l} C_{\tau }=f_{\tau }C_{\tau -1}+i_{\tau }{\tilde{C}}_{\tau } \end{array}$$

(24) $$\begin{array}{l} o_{\tau }=\sigma \left(\Theta_{o}\left[y_{\tau -1},{\tilde{x}}_{\tau }\right]+c_{o}\right) \end{array}$$

(25) $$\begin{array}{l} y_{\tau }=o_{\tau }\psi \left(C_{\tau }\right). \end{array}$$

and set ${ỹ}_{t}\left({\tilde{\theta }}^{LF}\right)=y_{t}$, where ${\tilde{\theta }}^{LF}$ contains the corresponding LSTM cell weights and intercepts. Using an LSTM cell circumvents the problem of lag order selection through either information criteria or shrinkage methods. The LSTM cell takes into account the whole sequence of ${\tilde{x}}_{1:t}$ by storing the necessary information in the memory cell. Figure 1 depicts the underlying idea.

We then linearly combine the output *ỹ*_*t*_ (for readability, we omit the dependence of *ỹ*_*t*_ on ${\tilde{\theta }}^{LF}$ from here on) with the other LF measures under consideration. This results in the following, case dependent, transformed HF information input variable


(26) $$\begin{array}{l} \nu_{t}^{HF}=\{\begin{array}{ll} {\tilde{x}}_{t} & \text{only recent HF information}\\ {ỹ}_{t} & \text{all HF information}. \end{array} \end{array}$$

We consider two settings, where we linearly combine the information from the past RV with that of the transformed HF returns and add an intercept. Herein, in resemblance to the classical HAR model, we first use past, daily, weekly, and monthly averages of the natural logarithm of RV (referred to as log RV hereafter). Denote the logarithm of RV at day *t* by ln *RV*_*t*_ i.e.,


(27) $$\begin{array}{l} \ln{RV}_{t}=\ln\left(RV_{t}\right). \end{array}$$

Weekly and monthly averages of log RV are then defined by


(28) $$\begin{array}{l} {\bar{\ln RV}}_{t}^{w}=\frac{1}{5}\overset{5}{\underset{i=1}{\sum }}\ln{RV}_{t-\left(i-1\right)} \end{array}$$

and


(29) $$\begin{array}{l} {\bar{\ln RV}}_{t}^{m}=\frac{1}{22}\overset{22}{\underset{i=1}{\sum }}\ln{RV}_{t-\left(i-1\right)}. \end{array}$$

Second, we use the output of an LSTM cell applied to the sequence of the past log RVs.

This results in the, case dependent, low frequency information input variable $\nu_{t}^{LF}$ and


(30) $$\begin{array}{l} \nu_{t}^{LF}=\{\begin{array}{l} {\left(\ln{RV}_{t},{\bar{\ln RV}}_{t}^{w},{\bar{\ln RV}}_{t}^{m}\right)}^{\prime }\\ LSTM\left(\ln{RV}_{1\;:\;t},\theta^{LF}\right) \end{array}. \end{array}$$

The HAR model is the most commonly used benchmark in volatility forecasting. However, its' implicit lag order selection (it is a restricted AR(22) model) is not necessarily validated in real data applications (Audrino and Knaus, 2016). As mentioned earlier, we circumvent the trouble of lag order selection since we apply an LSTM cell to the LF inputs. The LSTM cell can capture the long-term dynamics. Alternatively, one could fit an autoregressive model of order *p* on the RV, add the lags of the transformed measure as additional explanatory variables, and perform lag order selection via Information Criteria or shrinkage methods. However, we leave these two alternatives for further research.

We take the exponential of these linear combinations to guarantee the positiveness of the generated forecast. The output of the model thus is generated by


(31) $$\begin{array}{l} y_{t}\left(\theta \right)=\exp\left(c+{\left(\nu_{t}^{LF}\right)}^{\prime }\beta^{LF}+\beta^{HF}\nu_{t}^{HF}\right) \end{array}$$

where θ is a vector collecting all parameters. β^*LF*^ contains either the parameters associated with the daily, weekly, and monthly averages of log RV or the parameters associated with the output of the LSTM cell applied to the sequence of log RV. β^*HF*^ is the parameter of the scalar measure obtained from the transformation of the HF returns, and *c* is an intercept. The model that only uses the transformed HF returns for the forecast corresponds to restricting β^*LF*^ = 0. This comparison allows for a very detailed analysis of the source of potential gains in the forecasting performance:

1. We can assess whether there are significant differences in the forecasting performances of the models that only use the transformed measure as inputs to those that combine them with the LF variables. It is thus possible to inspect whether or not the sequence of transformed HF returns captures the information included in the past RV.
2. We can investigate whether it is necessary to consider the entire information in the transformed measure or whether the most recent information suffices.
3. We can compare the different transformation methods, assessing the differences between the linear MIDAS type transformations and the non-linear transformations.
4. We can examine whether using the classical HAR inputs with a fixed lag order of 22 is enough or whether using an LSTM cell on the past RV values, which is less restrictive in terms of the lag order selection, is fruitful.

### 5.1. Benchmark Models

We apply a variety of benchmark models, four models of the HAR family and an ARFIMA(p,d,q) model. Our proposed methodology ensures the positiveness of the volatility predictions by construction (see Equation 30). However, when fitting the benchmark models to the level of RV, the forecasts are not guaranteed to be positive. We thus implement each benchmark model once for the level of RV and once for the log of RV to allow for a fair comparison. In the latter case, the forecasts are bias corrected (Granger and Newbold, 1976), i.e.,


(32) $$\begin{array}{l} {\hat{RV}}_{t+1}=\exp\left({\hat{\ln RV}}_{t+1}+\frac{1}{2}\sigma_{\varepsilon }^{2}\right) \end{array}$$

where $\sigma_{\varepsilon }^{2}$ is the forecast error variance estimated from the residuals.

Following the suggestion of Andersen et al. (2003), we start by fitting an ARFIMA model


(33) $$\begin{array}{l} \left(1-\Phi \left(L\right)\right){\left(1-L\right)}^{d}x_{t}=\Theta \left(L\right)\varepsilon_{t},\text{ with }0<|d|<0.5 \end{array}$$

for *x*_*t*_ = *RV*_*t*_ and *x*_*t*_ = ln *RV*_*t*_, where ε_*t*_ is a Gaussian white noise with zero mean and variance σ^2^. Φ(*L*) and Θ(*L*) are lag polynomials of degrees *p* and *q*, respectively whose roots lie outside the unit circle.

Next, we implement benchmark models from the HAR family, starting with the classical HAR model (Corsi, 2009) in levels


(34) $$\begin{array}{l} RV_{t+1}=\beta_{0}+\beta_{1}RV_{t}+\beta_{2}{\bar{RV}}_{t}^{w}+\beta_{3}{\bar{RV}}_{t}^{m}+\varepsilon_{t+1} \end{array}$$

and in logs


(35) $$\begin{array}{l} \ln{RV}_{t+1}=\beta_{0}+\beta_{1}\ln{RV}_{t}+\beta_{2}{\bar{\ln RV}}_{t}^{w}+\beta_{3}{\bar{\ln RV}}_{t}^{m}+\varepsilon_{t+1}. \end{array}$$

For all HAR family models, the error term ε_*t*_ is assumed to be a white noise process with 𝔼[ε_*t*_] = 0 and $𝕍\left[\varepsilon_{t}\right]=\sigma_{\varepsilon }^{2}$. Following Andersen et al. (2007), we include the CHAR model as the second benchmark. The CHAR model is based on the jump robust Bi-Power Variation (BPV) measure of Barndorff-Nielsen and Shephard (2004), defined as


(36) $$\begin{array}{l} BPV_{t}=\frac{1}{\mu_{1}}\overset{M-1}{\underset{j=1}{\sum }}|r_{t,j}||r_{t,j+1}| \end{array}$$

where $\mu_{1}=\sqrt{2/\pi }$ is the expectation of the absolute value of a standard normal random variable. The CHAR model then replaces the daily, weekly, and monthly averages of RV on the right hand side of the HAR model with the corresponding averages of BPV, i.e., for levels


(37) $$\begin{array}{l} RV_{t+1}=\beta_{0}+\beta_{1}BPV_{t}+\beta_{2}{\bar{BPV}}_{t}^{w}+\beta_{3}{\bar{BPV}}_{t}^{m}+\varepsilon_{t+1} \end{array}$$

and for logs


(38) $$\begin{array}{l} \ln{RV}_{t+1}=\beta_{0}+\beta_{1}\ln{BPV}_{t}+\beta_{2}{\bar{\ln BPV}}_{t}^{w}+\beta_{3}{\bar{\ln BPV}}_{t}^{m}+\varepsilon_{t+1}. \end{array}$$

An alternative model that accounts for jumps is the HAR with jumps (HAR-J) model (Andersen et al., 2007). The HAR-J model adds the jump measure *J*_*t*_ = max(*RV*_*t*_ − *BPV*_*t*_, 0) or, when modeling log RV, ln (1 + *J*_*t*_), as an additional explanatory variable to the HAR model. However, in our application, the HAR-J model in levels produces negative volatility predictions in two cases. For the log case the average losses of the HAR-J model are very similar to those from the HAR model. We thus omit the results from the HAR-J model, though the differences are statistically significant. They are available from the authors upon request. The next benchmark model is the Semivariance-HAR (SHAR) model by Patton and Sheppard (2015), which builds on the semi-variation measure of Barndorff-Nielsen et al. (2010) differentiating between variation associated with positive and negative intraday returns. The estimators are defined as


(39) $$\begin{array}{l} RS_{t}^{+}=\overset{M}{\underset{j=1}{\sum }}r_{t,j}^{2}{𝕀}_{r_{t,j}>0} \end{array}$$

and


(40) $$\begin{array}{l} RS_{t}^{-}=\overset{M}{\underset{j=1}{\sum }}r_{t,j}^{2}{𝕀}_{r_{t,j}<0}, \end{array}$$

where 𝕀 is the indicator function and $RV_{t}=RS_{t}^{+}+RS_{t}^{-}$. The SHAR model uses this decomposition of *RV*_*t*_ such that for levels


(41) $$\begin{array}{l} RV_{t+1}=\beta_{0}+\beta_{1}^{+}RS_{t}^{+}+\beta_{1}^{-}RS_{t}^{-}+\beta_{2}{\bar{RV}}_{t}^{m}+\beta_{3}{\bar{RV}}_{t}^{m}+\varepsilon_{t+1} \end{array}$$

and for logs


(42) $$\begin{array}{l} \ln{RV}_{t+1}=\beta_{0}+\beta_{1}^{+}\ln{RS}_{t}^{+}+\beta_{1}^{-}\ln{RS}_{t}^{-}+\beta_{2}{\bar{\ln RV}}_{t}^{w}\\ \;+\beta_{3}{\bar{\ln RV}}_{t}^{m}+\varepsilon_{t+1}. \end{array}$$

The last benchmark from the HAR family is the HARQ model of Bollerslev et al. (2016). The HARQ model uses the Realized Quarticity (RQ) estimator of Barndorff-Nielsen and Shephard (2002a) to correct for measurement error in the RV estimator. The HARQ model for levels is


(43) $$\begin{array}{l} RV_{t+1}=\beta_{0}+\beta_{1}RV_{t}+\beta_{1Q}RQ_{t}^{1/2}RV_{t}+\beta_{2}{\bar{RV}}_{t}^{m}+\beta_{3}{\bar{RV}}_{t}^{m}+\varepsilon_{t+1} \end{array}$$

and for logs.


(44) $$\begin{array}{l} \ln{RV}_{t+1}=\beta_{0}+\beta_{1}\ln{RV}_{t}+\beta_{1Q}\ln{RQ}_{t}\ln{RV}_{t}+\beta_{2}{\bar{\ln RV}}_{t}^{w}\\ \;+\beta_{3}{\bar{\ln RV}}_{t}^{m}+\varepsilon_{t+1}. \end{array}$$

## 6. Application

We use the 5 minutes log-returns (*M* = 78 intraday observations per trading day) of IBM from January, 02, 2001 till December 28, 2018 (*T* = 4, 482 days). We use the first 80% of the data (till May 27, 2015) as the in-sample data and the last 20% as the out-of-sample data. In order to obtain forecasts from each model introduced earlier, the QLIKE loss (Patton, 2011) between the forecast *y*_*t*_(θ) and the next periods RV, *RV*_*t*+1_ is minimized, i.e., the objective is to find


(45) $$\begin{array}{l} \hat{\theta }={\mathrm{argmin}}_{\theta }\mathrm{QLIKE}\left(y_{t}\left(\theta \right),RV_{t+1}\right), \end{array}$$

where the QLIKE loss function is defined as


(46) $$\begin{array}{l} \mathrm{QLIKE}\left(RV_{t+1},y_{t}\left(\theta \right)\right)=\frac{RV_{t+1}}{y_{t}\left(\theta \right)}-\ln\left(\frac{RV_{t+1}}{y_{t}\left(\theta \right)}\right)-1. \end{array}$$

The QLIKE is a better choice when forecasting volatility than the mean squared error since it considers that the variable of interest is positive. We implement all models (except the benchmark models) in *Python* using *Keras* (Chollet, 2015) with the *TensorFlow* (Abadi et al., 2015) backend. This workflow comes with a comprehensive set of functions, allowing custom types of neural networks. We implement, e.g., the Beta MIDAS model as a specific case of an MLP that takes a 2 × 1 vector of ones as inputs and has a diagonal weight matrix coinciding with the parameters φ_1_ and φ_2_ of the Beta pdf. The layer then returns an *M* × 1 vector of weights associated with the standardized Beta pdf as described earlier. We estimate the parameters of all models under consideration (except the Benchmark models) by Stochastic Gradient Descent (SGD). Since SGD introduces an implicit regularization of the parameters (Soudry et al., 2018) this methodology should allow for a fair comparison of the forecasting results of the different models.

We train by Adaptive Moments SGD (ADAM, Kingma and Ba, 2014) with a batch size (length of a randomly selected sample selected for one SGD parameter update) of 128. *Keras* computes the gradient of RNNs by Truncated Back Propagation Through Time (Rumelhart et al., 1986); truncated in the manner that the computation of the gradient considers only a limited amount of past lags. The horizon of truncation is referred to as *lookback* and does not change the fact that the RNN considers the whole sequence of inputs when producing the forecast after training. We set the *lookback* equal to 128. We standardize the input data and divide the target data (the one step ahead RV) by its' standard deviation. We do not demean the target data to ensure positivity. We store the standard deviations to re-scale the resulting predictions in each forecasting step.

We use an expanding window scheme for forecasting: We start training for 1,000 epochs (one epoch means the algorithm went through the whole sample once) on the first 80% of the data and use the trained network and the newly available information to make a one step ahead prediction. Then, the model is re-trained for another 100 epochs for each one step ahead prediction with the previous iterations parameter values as starting values, resulting in 897 out of sample forecasts. To make training more feasible, we employ early stopping criteria. These interrupt the training before the target number of epochs is hit, given that there was no improvement of the training error over several specified past epochs. The term *patience* refers to this specified number of epochs. We set the minimum, absolute change of the training loss to be considered an improvement to 10^−6^, the initial training step *patience* to 500, and the *patience* in the re-training steps to 50. The code runs on an *NVIDIA Tesla V100* GPU on the bwHPC Cluster.

We estimate the HAR family benchmark models by OLS and the ARFIMA models using *R*'s *fracdiff* (Maechler, 2020) and *forecast* (Hyndman and Khandakar, 2008) packages. On the in-sample data, an ARFIMA(5,d,2) model provides the best fit for the level of RV and an ARFIMA (0,d,1) for the logarithm of RV.

### 6.1. Forecast Evaluation

We compare the forecasting performance of our presented model with varying inputs and the benchmark models for both levels and logs using different loss measures. First, we compare the average QLIKE loss of the different models. Next, we report the square root of the average squared error loss ${\left(RV_{t}-{\hat{RV}}_{t}\right)}^{2}$ (the RMSE). We further compute Value at Risk (VaR) and Expected Shortfall (ES) forecasts based on the volatility forecasts. The VaR is the *p*-th quantile of the return distribution and the ES is the expected value of the return, given that the return is smaller than the VaR. We compute the daily log returns *r*_*t*_ as the sum of the intraday returns of day *t*, which is equivalent to the log return based on the difference between the log closing and opening prices. Table 1 reports descriptive statistics of the daily returns (*r*_*t*_), the RV estimated from 5 minutes log-returns (*RV*_*t*_), and the standardized returns $z_{t}=r_{t}/\sqrt{RV_{t}}$ for the whole sample, the in- and the out-of-sample period.

**Table 1:** Descriptive statistics.

	**Min**	**Max**	**Mean**	**Median**	**Std**	**Skewness**	**Kurtosis**
**Whole-sample period**							
*r*_*t*_	−11.1695	11.6993	0.0053	0.0120	1.4997	0.1136	10.5653
*RV*_*t*_	0.219	130.5922	2.3877	1.0188	5.7425	10.0287	153.5745
*z*_*t*_	−2.9571	3.2444	0.0308	0.0142	0.9554	0.0833	2.6061
**In-sample period**							
*r*_*t*_	−11.1695	11.6993	0.0236	0.0148	1.5545	0.2314	10.1870
*RV*_*t*_	0.1325	130.5922	2.6083	1.1267	6.0041	9.9336	151.2284
*z*_*t*_	−2.9571	3.2444	0.0408	0.0147	0.9514	0.0910	2.6362
**Out-of-sample period**							
*r*_*t*_	−7.9331	8.5542	−0.0679	0.0000	1.2548	−0.8824	11.5016
*RV*_*t*_	0.1219	63.4346	1.5063	0.6800	4.4410	9.7437	113.8447
*z*_*t*_	−2.5287	2.7506	−0.0092	0.0000	0.9703	0.0594	2.4857

*For the descriptive statistics, r_t_ is scaled by 10^2^ and RV_t_ by 10^4^*.

After standardizing the daily returns, their skewness and kurtosis are close to those of a standard normal distribution. For the out-of-sample period, we can not reject the H0 of a Kolmogorov-Smirnov test that the standardized returns are standard normally distributed (*p*-value = 0.646). For the whole sample (*p*-value = 0.018) and the in-sample period (*p*-value = 0.011) we reject this hypothesis at the 5% level. These results are reasonable since the out-of-sample period does not contain the financial crisis. We thus use the normal distribution to compute forecasts of VaR and ES. We also compute forecasts of VaR and ES using the standardized Student-t distribution, where, similar to Brownlees and Gallo (2010), for each iteration in the expanding window, we estimate the degrees of freedom based on the information available up to time *t*. All estimated degrees of freedom are larger than 100, indicating no need to account for fat tails. Further, the statistical analysis results and the ranking of the models do not change compared to the case of the normal distribution. We thus do not report the Student-t distribution results here. They are available from the authors on request.

To evaluate the performance of the models in forecasting VaR and ES, we use the *asymmetric piece-wise linear* loss function of Gneiting (2011) for the VaR and the *zero-homogeneous* loss function of Fissler and Ziegel (2016) for the VaR and the ES jointly.^3^ Using the short notation *r* = *r*_*t*_, $\hat{VaR}={\hat{VaR}}_{t,p}$ and $\hat{ES}={\hat{ES}}_{t,p}$, these loss functions are


(47) $$\begin{array}{l} S_{p}^{VaR}\left(\hat{VaR},r\right)=\left(r-\hat{VaR}\right)\left(p-{𝕀}_{\left\{r\leq \hat{VaR}\right\}}\right) \end{array}$$

and.


(48) $$\begin{array}{l} S_{p}^{VaRES}\left(\hat{VaR},\hat{ES},r\right)=-\frac{\left(\hat{VaR}-r\right){𝕀}_{\left\{r\leq \hat{VaR}\right\}}}{p\hat{ES}}+\frac{\hat{VaR}}{\hat{ES}}+\ln\left(-\hat{ES}\right)-1. \end{array}$$

### 6.2. Results

Table 2 reports the results of the out-of-sample losses introduced above for the different models. It further shows which models are in the Model Confidence Set (MCS) of Hansen et al. (2011) at the 10% level. We use the *arch* library of Sheppard et al. (2021) to compute the MCS *p*-values. In addition, we report the results of Binomial tests,^4^ where we test each model against the other models in Supplementary Figures 1–6. The table consists of two main blocks, again consisting of multiple blocks as indicated by the horizontal lines. The first main block contains the results for the ANN models, where the first two rows show the results for the models that do not use the transformed measure as additional input (indicated by the superscript O), i.e., the models corresponding to the restriction β^*HF*^ = 0. The first model is a non-linear HAR estimated by SGD. Non-linear since we use the exponential of a linear combination of daily, weekly, and monthly averages of log RV on the right-hand side. The second row shows the results of modeling the long memory in RV not via the restricted AR(22) character of the HAR model but an LSTM cell applied to the log RV.

**Table 2:** Out of sample losses.

	**Model**	**QLIKE**	**RMSE**	**VaR_**1*%***_**	**VaR ES_**1*%***_**	**VaR_**2.5*%***_**	**VaR ES_**2.5*%***_**
**ANN models**	HAR^O^	0.6012^†^	0.4401	0.6598^†^	−2.2635^†^	1.0444^†^	−3.0502^†^
	LSTM^O^	0.5683^†^	0.4356^†^	0.6687^†^	−2.1732^†^	1.0497^†^	−3.0029^†^
	HAR^M−B^-F	0.5995^†^	0.4407^†^	0.6580^†^	−2.2793^†^	1.0374^†^	−3.0671^†^
	HAR^M−L^-F	0.5655^†^^*^	0.4380^†^^*^	0.6397^†^	−2.3329^†^	1.0167^†^	−3.1032^†^
	HAR^LSTM1^-F	0.5990^†^^*^	0.4347^†^^*^	0.6446^†^^*^	−2.2779^†^^*^	1.0278^†^^*^	−3.0626^†^^*^
	HAR^LSTM8^-F	0.5976^†^	0.4400	0.6576^†^	−2.2711^†^	1.0420^†^	−3.0545^†^
	HAR^LSTM64^-F	0.5992^†^	0.4400	0.6579^†^	−2.2731^†^	1.0420^†^	−3.0559^†^
	LSTM^M−B^-F	0.5813^†^	0.4361^†^	0.6616^†^^*^	−2.2074^†^^*^	1.0462^†^^*^	−3.0209^†^^*^
	LSTM^M−L^-F	0.5458^†^	0.4354^†^	0.6617^†^	−2.2360^†^	1.0378^†^	−3.0464^†^
	LSTM^LSTM1^-F	0.5779^†^	0.4406^†^	0.6668^†^^*^	−2.1577^†^^*^	1.0513^†^^*^	−2.9970^†^^*^
	LSTM^LSTM8^-F	0.5701^†^	0.4356^†^	0.6688^†^	−2.1669^†^	1.0508^†^^*^	−3.0011^†^^*^
	LSTM^LSTM64^-F	0.5693^†^	0.4356^†^	0.6626^†^^*^	−2.2115^†^^*^	1.0479^†^^*^	−3.0200^†^^*^
	O^M−B^-F	0.7923^*^	0.5205^†^^*^	0.6666^†^^*^	−2.5151^†^^*^	1.1599^†^^*^	−3.0053^†^^*^
	O^M−L^-F	0.7035^†^^*^	0.4499^†^^*^	0.7301^†^^*^	−2.1101^†^^*^	1.1650^†^^*^	−2.8801^†^^*^
	O^LSTM1^-F	0.7468^*^	0.4514^*^	0.6772^†^^*^	−2.4570^†^^*^	1.1580^*^	−2.9845^†^^*^
	O^LSTM8^-F	0.7536^*^	0.4517^*^	0.6695^†^^*^	−2.4596^†^^*^	1.1496^*^	−2.9898^†^^*^
	O^LSTM64^-F	0.7458^*^	0.4516^*^	0.6525^†^^*^	−2.5659^†^^*^	1.1448^†^^*^	−3.0239^†^^*^
	HAR^M−B^	0.6108	0.4403	0.6613^†^	−2.2538^†^	1.0485^†^	−3.0407^†^
	HAR^M−L^	0.5764^†^	0.4406^†^	0.6620^†^	−2.2263^†^	1.0456^†^	−3.0356^†^
	HAR^LSTM1^	0.5453^†^^*^	0.4339^†^^*^	0.6224^†^^*^	−2.4520^†^^*^	**0**.**9901**^†^^*^	**−3**.**1643**^†^^*^
	HAR^LSTM8^	0.6029	0.4400	0.6576^†^	−2.2768^†^	1.0401^†^	−3.0606^†^
	HAR^LSTM64^	0.5989^†^	0.4400	0.6594^†^	−2.2576^†^	1.0448^†^	−3.0472^†^
	LSTM^M−B^	0.5764^†^^*^	0.4356^†^^*^	0.6627^†^^*^	−2.2073^†^^*^	1.0508^†^^*^	−3.0143^*^
	LSTM^M−L^	0.5567^†^	0.4350^†^	0.6648^†^	−2.1684^†^	1.0413^†^	−3.0117^†^
	LSTM^LSTM1^	**0**.**5371**^†^^*^	**0**.**4316**^†^^*^	**0**.**6193**^†^^*^	−2.4187^†^^*^	0.9993^†^^*^	−3.1350^†^^*^
	LSTM^LSTM8^	0.5755^†^	0.4352^†^	0.6631^†^	−2.1964^†^	1.0445^†^	−3.0176^†^
	LSTM^LSTM64^	0.5712^†^^*^	0.4361^†^^*^	0.6656^†^^*^	−2.2058^†^^*^	1.0504^†^^*^	−3.0169^†^^*^
	O^M−B^	0.7463^*^	0.4525^*^	0.6494^†^^*^	−2.5692^†^^*^	1.1381^†^^*^	−3.0315^†^^*^
	O^M−L^	0.7480^*^	0.4529^*^	0.6515^†^^*^	−2.5576^†^^*^	1.1389^†^^*^	−3.0276^†^^*^
	O^LSTM1^	0.6701^†^^*^	0.4466^*^	0.6333^†^^*^	**−2**.**5970**^†^^*^	1.0973^†^^*^	−3.0812^†^^*^
	O^LSTM8^	0.7413^*^	0.4517^*^	0.6519^†^^*^	−2.5700^†^^*^	1.1396^*^	−3.0323^†^^*^
	O^LSTM64^	0.7457^*^	0.4516^*^	0.6525^†^^*^	−2.5681^†^^*^	1.1456^†^^*^	−3.0240^†^^*^
**Benchmark models**	ARFIMA	0.6947^*^	0.4538^*^	0.6906^†^^*^	−2.1262^†^^*^	1.0833^†^^*^	−2.9677^†^^*^
	HAR	0.6758^*^	0.4532^*^	0.6975^†^^*^	−2.1380^*^	1.0925^†^^*^	−2.9620^†^^*^
	CHAR	0.5665^†^^*^	0.4384^†^^*^	0.6462^†^^*^	−2.3974^†^^*^	1.0455^†^^*^	−3.0830^†^^*^
	SHAR	0.6785^*^	0.4548^*^	0.6976^†^^*^	−2.1348^*^	1.0928^†^^*^	−2.9613^†^^*^
	HARQ	0.6632^*^	0.4525^*^	0.6827^†^^*^	−2.1610^*^	1.0736^†^^*^	−2.9881^†^^*^
	ARFIMA-ln	0.6656^*^	0.4394^†^^*^	0.7095^†^^*^	−1.9656^*^	1.0812^†^^*^	−2.9242^†^^*^
	HAR-ln	0.6751^*^	0.4396^†^^*^	0.7020^†^^*^	−2.0047^†^^*^	1.0703^†^^*^	−2.9496^†^^*^
	CHAR-ln	0.6141^*^	0.4373^†^^*^	0.6773^†^^*^	−2.1527^†^^*^	1.0503^†^^*^	−3.0169^†^^*^
	SHAR-ln	0.6549^*^	0.4389^†^^*^	0.6922^†^^*^	−2.0547^†^^*^	1.0615^†^^*^	−2.9743^†^^*^
	HARQ-ln	0.6413^*^	0.4403^†^^*^	0.6930^†^^*^	−2.0982^†^^*^	1.0660^†^^*^	−2.9873^†^^*^

*The RMSE and VaR losses are scaled by 10^3^. Bold face numbers indicate the lowest out of sample loss*.

* *Denotes models for which the H_0_ of equal forecasting performance of a Binomial test with HAR^O^ model as benchmark is rejected at the 5% level and*

† *denotes models that are in the Model Confidence set at the 10% level*.

Next, follow the models that use the information from the transformed measure. The superscript indicates the type of transformation: The superscript O indicates no transformation, the superscripts M-B and M-L indicate the Beta and LSTM MIDAS transformation, respectively, and the superscript LSTM plus a number indicates the non-linear, LSTM based transformation. The number indicates the number of LSTM cells in the hidden layer in this case.

The model's name indicates the type of low-frequency information: HAR refers to the daily, weekly, and monthly averages, and LSTM refers to an LSTM cell applied to the sequence of log RV. They reflect choosing $\nu_{t}^{LF}={\left(\ln{RV}_{t},{\bar{\ln RV}}_{t}^{w},{\bar{\ln RV}}_{t}^{m}\right)}^{\prime }$ and $\nu_{t}^{LF}=LSTM\left(\ln{RV}_{1:t},\theta^{LF}\right)$ respectively. The name O refers to only using the information from the transformed measure, i.e., it corresponds to the restriction β^*LF*^ = 0. Here we have two blocks again. The first reporting models that apply an LSTM cell to the sequence of the transformed measure. These models thus take into account the full information in the sequence of the transformed measure, indicated by -F in the model name. The second block refers to models that only use the most recent value of the transformed measure. The two blocks thus correspond to the choice of $\nu_{t}^{HF}={\tilde{x}}_{t}$ and $\nu_{t}^{HF}={ỹ}_{t}$, respectively. The last block shows the results of the benchmark models, where we differentiate between them being applied for the level of RV and to the logarithm of RV (indicated by -ln in the model name). We transform the forecasts using the bias correction mentioned earlier for the latter case.

**Figure 2:**
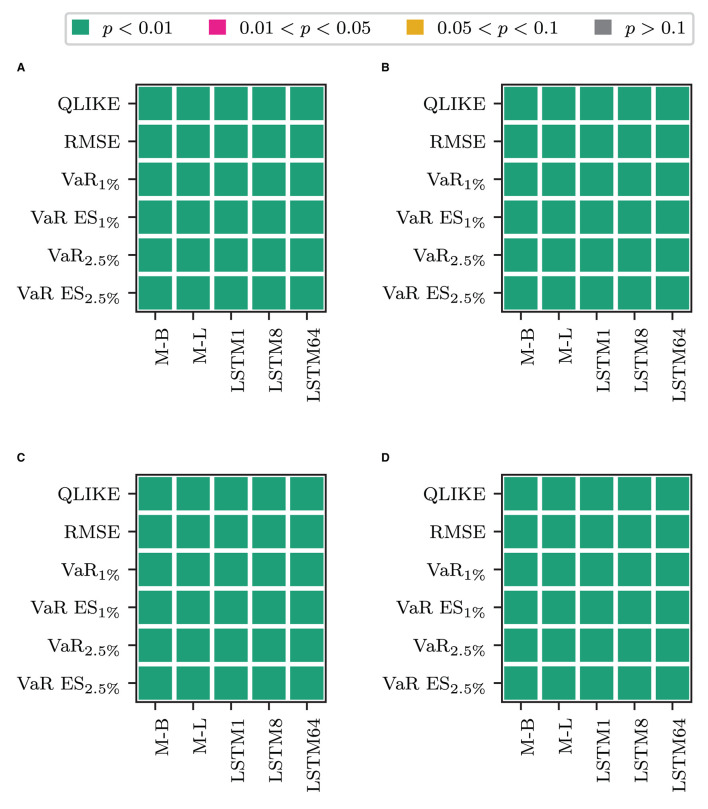
Results for a Binomial test of equal forecasting performance between the models that use only the transformed measure and their counterpart that use it in combination. **(A)** HAR-F vs. O-F. **(B)** HAR vs. O. **(C)** LSTM-F vs. O-F. **(D)** LSTM vs. O.

We first consider whether using only the transformed measure for forecasting volatility is fruitful. Table 2 clearly shows that the models that only rely on the transformed measure (labeled O plus the superscript corresponding to the transformation used) are the worst-performing models within their respective blocks in terms of the QLIKE and the squared error loss. These models perform comparably or worse than the alternatives for the VaR loss and the joint loss of VaR and ES. The only exception is when jointly evaluating forecasts of VaR and ES at *p* = 1%. In this case, these models are the best performing ones, and among them, the model that uses the non-linear transformation via one LSTM cell performs best. The differences in the forecasting performance of the only transformed measure models to those that also use the information on past RV (HAR and LSTM plus superscript) are significant in terms of a binomial test for equal forecasting performance at the 1% level, as Figure 2 shows. Figure 2A of the figure displays the test decision when comparing the models that combine the HAR inputs with *ỹ*_*t*_ against the model that only uses *ỹ*_*t*_ (O plus superscript). The x-axis labels specify the type of transformation used to obtain the transformed measure. Figure 2B shows the results for only using the most recent information in the transformed measure, i.e., combining the HAR inputs with ${\tilde{x}}_{t}$ vs. solely using ${\tilde{x}}_{t}$. Figures 2C,D show the results for the case where the HAR inputs are replaced by the output of an LSTM cell applied to the sequence of log RV. All *p*-values are smaller than 0.01 in all cases. For the QLIKE and the squared error loss, we can conclude that none of the transformations can extract enough information from the HF returns to replace the information on past RV for forecasting volatility. When forecasting the VaR and ES, these models yield results comparable to those of the other models. They outperform the alternative models only for the joint evaluation of the VaR and the ES at the 1% level.

**Figure 3:**
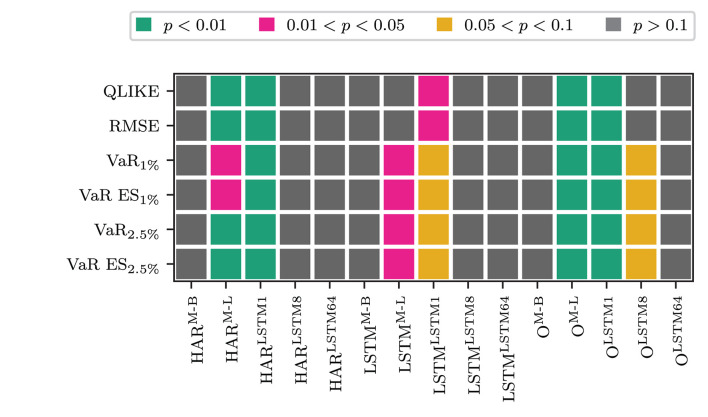
Results for a Binomial test of equal forecasting performance between the full information (*ỹ*_*t*_) and the recent information (${\tilde{x}}_{t}$) models.

Next, we address whether the -F models (the models that use the output of an LSTM cell applied to the sequence of the transformed measure) yield any differences in the forecasting performance compared to the models that only use the most recent information from the transformation. Figure 3 shows the testing results for differences between a model that only uses the most recent information in the transformed measure against its' -F counterpart. At the 5% level, regardless of the LF input they are combined with, we see no significant differences in the forecasting performance of the MIDAS Beta, the LSTM8, and the LSTM64 transformation models compared to their -F counterparts. However, these differences are significant for the LSTM MIDAS and the LSTM1 transformation. When we only use the transformed measure, the LSTM MIDAS transformation model with full information produces lower average OLIKE and squared error losses. In contrast, the model that only uses the most recent information produces lower average losses when jointly evaluating VaR and ES. For the LSTM1 transformation, the model that only uses the most recent information yields the lower average loss for all loss functions. Combining the transformed measure with other LF information yields the following pattern: For the LSTM MIDAS transformation, where the differences are significant, the -F model gives the lower average losses. For the LSTM1 transformation, using only the most recent information yields lower average losses.

Whether there are significant differences in the forecasting performance between the models that use *ỹ*_*t*_ and the models that use ${\tilde{x}}_{t}$ is thus case dependent. There are no significant differences for most models and transformations. For the LSTM MIDAS, it depends on whether it is used alone or in combination. In the former case, the -F models produce lower losses when the differences are significant. Using the -F models yields the lower QLIKE and squared error in the latter case. However, using ${\tilde{x}}_{t}$ produces lower VaR and ES losses. For the non-linear transformation through one LSTM cell, only applying the transformation to the most recent HF returns yields lower average losses. It seems that, in this case, the more distant information in the HF returns gets accounted for by the RV. However, the most recent HF returns contain information that the lagged RV does not yet capture.

When we use the LSTM MIDAS transformation, it is necessary to use the sequential information in the transformed measure. An alternative explanation for this could be that the -F model introduces additional non-linearity into the transformed measure by applying an LSTM cell to its' sequence. While ${\tilde{x}}_{t}$ in the LSTM MIDAS case is constructed linearly as a weighted sum, *ỹ*_*t*_ is a non-linear transformation of the sequence of that linear measure. So the better forecasting performance of the model that uses *ỹ*_*t*_ for the LSTM MIDAS transformation could be due to that non-linearity. However, the transformation of the HF returns through one LSTM cell is already the output of a non-linear function. Since only the most recent transformed measure is informative for this transformation, it appears that there are no gains from introducing more non-linearity through an LSTM cell on the sequence of transformed measures. Comparing these two against each other, we see that models that use only the most recent non-linear transformed measure produce lower losses than the models that use the LSTM cell applied to the LSTM MIDAS transformation. These differences are significant at the 1% level for all losses (see Supplementary Figures 1–6). Thus, the non-linearity within the transformed measure seems to produce more helpful information for forecasting volatility than introducing non-linearity to the transformed measure obtained from the linear method.

**Figure 4:**
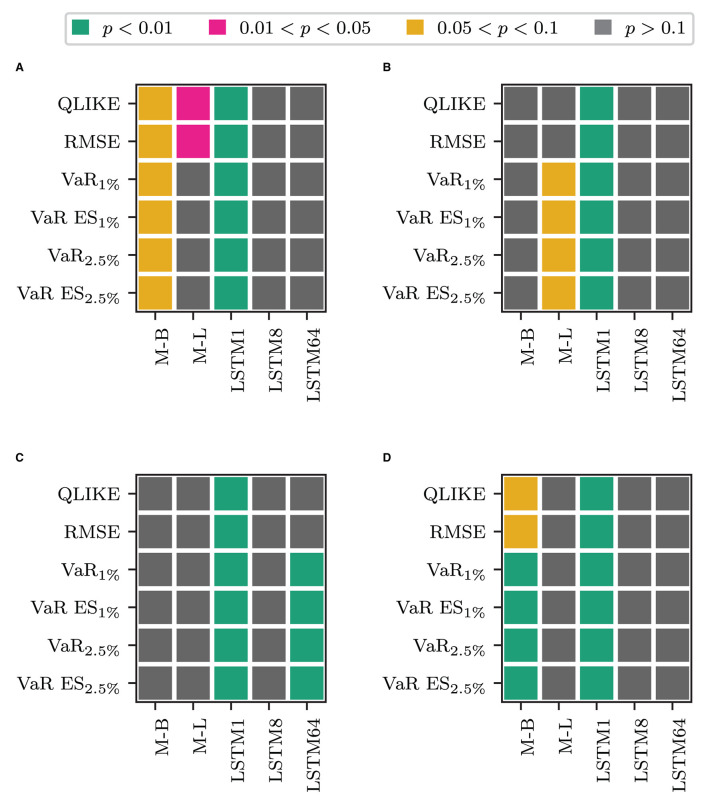
Results for a Binomial test of equal forecasting performance between the models combine the transformed measure and their counterpart that does not use the transformed measure. **(A)** HAR-F vs. HAR^O^. **(B)** HAR vs. HAR^O^. **(C)** LSTM-F vs. LSTM^O^. **(D)** LSTM vs. LSTM^O^.

Next, we address whether combining the transformed measure with the other LF inputs yields significant gains in forecasting compared to only using the LF inputs. Figure 4 displays the test results. Figures 4A,B show the test results for combining the HAR model inputs with the transformed measures against the model that does not use the transformation. The x-axis labels again indicate the type of transformation. Figure 4A shows the results for the -F models and Figure 4B for the models that only use the most recent information. The lower part of the figure, Figures 4C,D, display the corresponding results when replacing the HAR inputs with the output of an LSTM cell applied to the sequence of log RV. Combining any of the LF inputs with the LSTM1 transformation yields statistically different forecasts to the models that omit the transformed measure, in any case, and for all losses. In the case of the HAR model inputs, the combined model yields lower losses in both cases. For the LSTM input, the -F model performs worse, whereas the model that only uses the most recent HF information yields lower losses. For the other transformations, the results are case-dependent. When combined with the HAR inputs, the LSTM MIDAS model yields significantly different QLIKE and RMSE losses in the full information case. The HAR^M−L^-F model yields the lower QLIKE and RMSE losses in these two cases. The differences are not statistically different at the 5% level in the remaining cases. The non-linear transformation with 64 LSTM cells yields statically different results for all losses but the QLIKE and the squared error loss in the full information case. Its' losses are lower than the comparison model in these cases. When using the recent information only the non-linear transformation with 64 LSTM cells does not deliver significantly different results. However, in the full information case, the Beta MIDAS transformation for the VaR and ES for *p* = 1% and *p* = 2.5% yields losses significantly different from the comparison model's (at the 5% level). In these cases, the Beta MIDAS transformation model yields slightly better results.

**Figure 5:**
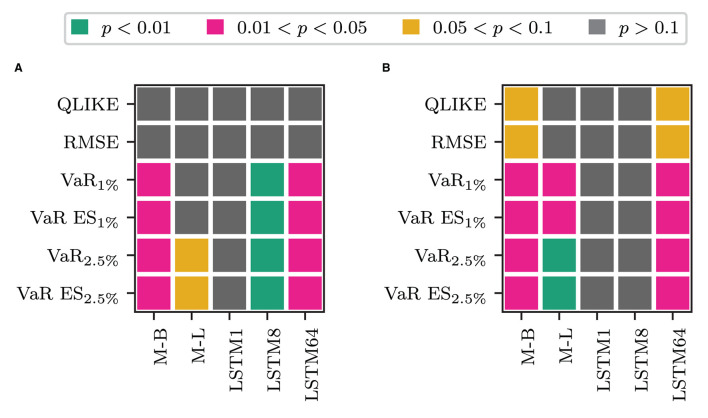
Results for a Binomial test of equal forecasting performance between the models that combine the transformed measure with the HAR inputs and their counterpart that uses the LSTM cell applied to log RV. **(A)** HAR-F vs LSTM-F. **(B)** HAR vs LSTM.

Next, we consider whether there are differences in the forecasting performances of the models depending on whether we use the HAR inputs or the output of an LSTM cell applied to past log RV. Table 2 reports a rejection of the H0 of the Binomial test for equal forecasting performance concerning the HAR^O^ model at the 5% level with an asterisk. From the table, we see that for the LSTM^O^ model, we can not reject the H0 for any of the loss measures. Thus there are no significant differences between the ANN model that only uses the HAR model inputs and the ANN model that uses an LSTM cell on past log RVs. One difference is that the LSTM^O^ model is in the MCS at the 10% level for all losses, whereas the HAR^O^ model is not in the 10% MCS for the squared error loss. For the remaining models that use the transformed measure, the test results are displayed in Figure 5.

According to the figure, we can not reject the H0 at the 5% level for the QLIKE and the squared error loss. For the VaR and ES losses, we reject the H0 at the 5% level for the Beta MIDAS, the LSTM8, and the LSTM64 transformations when using *ỹ*_*t*_. In this case, the models that use the HAR inputs perform better than those using the LSTM input. When using ${\tilde{x}}_{t}$, we reject for the Beta and LSTM MIDAS models and the LSTM64 models considering the VaR and ES losses. In all except one case, the models that use the HAR inputs yield the lower out of sample loss. The only exception is the LSTM MIDAS model for the VaR_2.5%_. In this case, the LSTM inputs are performing marginally better. Overall, it appears that the HAR model inputs can approximate the long memory in the data to an extent comparable to that of an LSTM cell. However, we want to stress that we did not hunt for an optimal LSTM network architecture for this task. The purpose of the LSTM cell in this application is simply to circumvent the implicit lag order selection of the HAR model. A network of LSTM cells applied to the sequence of log RV as in Bucci (2020) might yield more consistent improvements in the forecasting performance than the HAR model inputs. It is interesting to see that the daily, weekly, and monthly averages used in the HAR model are not only comparable to ARFIMA models in the extent they account for long memory (Corsi, 2009), but also to an LSTM cell.

Among the benchmark models, the CHAR model is performing best. It produces the lowest out of sample loss among the benchmark models for the level and the log of RV. Furthermore, it produces lower losses for all except the RMSE loss in levels than in logs. It is the only benchmark model in the 10% MCS for all losses and it produces lower QLIKE and RMSE losses than the HAR^O^ model, i.e., the HAR model estimated by SGD. These differences are significant at the 5% level. Also, for the other losses except for the VaR_2.5%_, it yields lower losses than the HAR^O^. This is in line with Rahimikia and Poon (2020a), who also find that the CHAR is performing best among the HAR family models. Apart from the CHAR model, the remaining benchmark models cannot perform better than any of the ANN models except those that only use the transformed measure.

We come to a short intermediate conclusion:

1. We found that using only the transformed measure to forecast RV results in higher out-of-sample forecast losses than models that combine the transformed measure with information on past log RV. This holds especially true for the QLIKE and the RMSE error loss. The only exception is the loss of jointly evaluating the VaR and ES at *p* = 1%.
2. We found that when using linear means to construct the transformed measure, it is crucial to consider the sequential information in the transformed measure. However, this might be due to non-linearity induced through the LSTM cell that we apply to the transformed measure. Therefore, it is sufficient only to use the most recent information when constructing the transformed measure non-linearly. In most cases, this yields better forecasting performance.
3. The non-linear transformation through one LSTM cell seems superior to the other transformations throughout the statistical analysis. The models performing best are those that use this transformation. Further, we have the most statistical evidence for differences in the forecasting performance for these models. We will further investigate this in the following.
4. For the QLIKE and the RMSE loss, there are no statistical differences in the performance of the models that use the HAR inputs and the models that use an LSTM cell applied to log RV. The daily, weekly, and monthly averages of log RV appear to be sufficient to account for the long memory in the data. Especially when combined with the LSTM1 transformed measure, this also holds for all other losses.

This short wrap-up leads to two hypotheses. First, the non-linear transformation through one LSTM cell is superior to all other transformations. Second, the models that combine the transformed measure from such a non-linear transformation with the information on past log RV perform better than all other models. These two models are the two best ranked models for each loss measure, except the joint evaluation of VaR_1%_ and ES_1%_. We cannot reject that these two models perform equally well for any of the losses (see Supplementary Figures 1–6).

Investigating these hypotheses results in non-pairwise comparisons of the models. Further, the hypotheses are uni-directional, i.e., we are interested in whether these models perform better than the competitors. Thus we can not use a Binomial Test for equal forecasting performance but instead use the test for superior predictive ability (SPA test) of Hansen (2005). We use the *arch* library of Sheppard et al. (2021) to perform the SPA test. When computing the p-values, we use a block bootstrap with the number of bootstrap resamplings set to 1000 and the block length set to 5. The results are not sensitive to the choice of these two values. We also computed the p-values with resamplings set to 3,000, 5,000, 7,000, 9,000 and block lengths of 10, 15, 20, …, 95, 100. The results did not change by much. The SPA test tests whether the expected loss difference between the loss of a candidate and a set of alternative models is smaller or equal to zero. A rejection of the null hypothesis thus means that there is a model among the alternatives performing significantly better than the candidate model.

We start by reporting the *p*-values of a sequence of SPA tests where we use the LSTM1 transformation models as candidates against the models that use the other transformations. The p-values displayed in Table 3 show that, at the 5% level, we can not reject the H0 of the SPA test in any case. Thus, at the 5% level, the non-linear transformation by one LSTM cell gives forecasting losses smaller or equal to those of all alternative transformations used. This holds for any loss function. At the more conservative 10% level, for the models that use the full information on the transformed measure (upper part of the table) and the joint loss of VaR and ES at 2.5%, we reject the H0. Thus, for this loss, at least one transformation works better. Overall, however, this evidence supports the first hypothesis of the non-linear transformation through one LSTM cell performing best.

**Table 3:** *p*-values of SPA tests for the LSTM1 against the alternative transformations.

	**QLIKE**	**RMSE**	**VaR_**1*%***_**	**VaR ES_**1*%***_**	**VaR_**2.5*%***_**	**VaR ES_**2.5*%***_**
HAR^LSTM1^-F	0.182	0.870	0.542	0.385	0.365	0.232
LSTM^LSTM1^-F	0.124	0.135	0.456	0.156	0.162	0.086
O^LSTM1^-F	0.233	0.724	0.405	0.431	0.603	0.517
HAR^LSTM1^	0.823	0.937	0.580	0.567	0.543	0.546
LSTM^LSTM1^	0.707	0.967	0.607	0.578	0.548	0.591
O^LSTM1^	0.543	0.593	0.906	0.789	0.601	0.975

To assess the second hypothesis, we use all models excluding the HAR^LSTM1^ and LSTM^LSTM1^ as the set of alternatives. We then apply the SPA test for each of these two models as candidates. Table 4 displays the *p*-values of those tests. Again, we see that the null hypothesis that no alternative model performs better than any of the two models under consideration can not be rejected for any loss function. Among the considered models, including the benchmarks for logs and levels, no model performs significantly better than the HAR^LSTM1^ and the LSTM^LSTM1^.

**Table 4:** *p*-values of SPA tests for HAR^LSTM1^ and LSTM^LSTM1^ against the remaining models.

	**QLIKE**	**RMSE**	**VaR_**1*%***_**	**VaR ES_**1*%***_**	**VaR_**2.5*%***_**	**VaR ES_**2.5*%***_**
HAR^LSTM1^	0.726	0.952	0.975	0.527	0.955	0.996
LSTM^LSTM1^	0.794	0.993	0.970	0.408	0.928	0.973

## 7. Conclusion

This paper aims to forecast the daily volatility utilizing information extracted from the intraday high-frequency (HF) returns through Long Short Term Memory (LSTM) Recurrent Neural Networks (RNN). These structures are flexible in the degree of non-linearity they allow for and capture long persistence in the data. Our method extracts a non-linear, scalar transformation of the HF returns (referred to as transformed HF measure). We use this measure to make one step ahead predictions of the daily volatility. We vary the degree of non-linearity by testing different numbers of LSTM cells in the RNN and find no merits in using more than one LSTM cell for the non-linear transformation. For comparison, we implement two Mixed Data Sampling (MIDAS) approaches to construct the transformation of the HF returns. The MIDAS models obtain weights associated with the HF return and build the transformation as a weighted sum. The first MIDAS model generates weights associated with the lag of an intraday return through an LSTM cell (LSTM MIDAS). The second is an Artificial Neural Network (ANN) implementation of the Beta Lag Polynomial MIDAS (Beta MIDAS) (Ghysels et al., 2004).

To account for dynamics and long memory in the volatility series, we apply an LSTM cell to the sequence of transformed measures. However, we also compare settings where we only use the most recent information from the transformed measure. The reason is that the information from the HF returns might only be “new” for a short time. Further in the past, it is probably incorporated by the RV estimator. We compare the forecasting performance of models solely based on the transformed HF measure to those of models that only use the information from the past Realized Volatility (RV). Namely, the HAR model and a model that applies an LSTM cell to the sequence of past RVs. The HAR model is one of the most popular models to approximate long memory in the volatility series. LSTM RNNs can account for complex non-linear dependencies in the data and capture long-term dependencies. Our comparison assesses whether the proposed transformation can extract the same or more information from the HF returns than the RV estimator. Finally, we combine the information from the transformed measure and the information from the RV for the forecast. We can thus investigate whether our proposed transformations extract information from the HF returns that is supplementary to the RV information when forecasting volatility.

In an expanding window forecasting exercise on data on the IBM stock, we compare the performance of the models in forecasting out-of-sample volatility. We further compute Value at Risk (VaR) and Expected Shortfall (ES) forecasts based on the volatility forecast. We perform a thorough statistical analysis to identify the source of the improved forecasting performance. Our results on the data set under consideration are four-fold:

First, they show that making volatility forecasts based solely on the transformed HF measure is not fruitful. Neither of the transformations can produce a measure that accounts for the long persistence in the volatility. This result is independent of whether we account for dynamics in the transformed measure or only take the most recent value for the forecast. Interestingly, when jointly evaluating the VaR_1%_ and ES_1%_ forecasts based on the volatility forecasts, those models perform better than the alternatives. However, for the 2.5% VaR and ES, their performance is again worse or comparable to the alternatives. When forecasting volatility, the transformations we propose are thus unable to extract the same information from the high-frequency returns as the RV estimator. Since the RV estimator ex-post is a consistent estimator of the volatility of a day, it is crucial to take this information into account for the forecasting task. Maybe more complex non-linear ANN structures could extract the same amount of information from the HF returns. However, in our eyes, it is more fruitful to facilitate the forecasting task for the method by using the RV information.

Second, there is no difference in using the sequence of the transformed measure or only the most recent value for most cases. There are significant differences only for the LSTM MIDAS transformation and the non-linear transformation based on one LSTM cell. The LSTM MIDAS transformation excels when we account for dynamics in the transformed measure. In contrast, the non-linear transformation excels when only using the most recent information. Though puzzling at first, this finding is quite intuitive. The LSTM MIDAS builds the transformed measure as a weighted sum. The transformation is thus linear. However, the linearity is insufficient to extract additional information from the HF returns. Therefore the model that only uses the most recent transformed measure, in this case, performs no different than the model that does not use the information. However, we account for dynamics in the sequence of transformed measures by applying an LSTM cell to it. While this circumvents the trouble of lag order selection, it introduces non-linearity in the transformed measure, which likely results in better models' better performance. When we use an LSTM cell to transform the HF returns non-linearly, there are no additional gains from accounting for dynamics in the measure. Accounting for dynamics leads to worse forecasting performance in some cases. We thus conclude that the transformed measure must be non-linear for the transformation to extract additional information from the HF returns. However, allowing for dynamics in the non-linearly obtained transformed measure does not add any additional gains. This coincides with our previous findings indicating that the additional information in the HF returns gets picked up by the RV estimator further in the past. In the short run, though, this information is helpful for the prediction of volatility.

Third, we add to the literature by finding another prove for the improved forecasting performance of ANN models compared to the linear HAR model benchmark. Our models that do not include the transformed measure, i.e., only use either the HAR model inputs or apply an LSTM cell to the sequence of RV, perform significantly differently from the classical HAR model, both estimated in logs and levels. The simple non-linearity we induce through modeling the exponential of the linear combination of past daily, weekly, and monthly averages of the logarithm of RV already is sufficient to outperform the classical linear HAR for both logs and levels. Our results thus add to the evidence provided by, e.g., Rosa et al. (2014) and Arnerić et al. (2018). We also apply an LSTM cell to the sequence of the logarithm of RV as an alternative to the HAR inputs. The LSTM cell allows for a high degree of non-linearity, and it captures long memory in the data. We find no significant differences between the LSTM and the HAR input models when predicting volatility in most cases. Our findings thus indicate that, for the simple structures we use, the HAR inputs capture the long persistence in the volatility series equally well as the LSTM cell on the data set under consideration. To some extent, this contradicts the findings of Bucci (2020) who finds that gated recurrent ANNs such as LSTM RNNs outperform ANNs that do not account for long memory in the data. However, the author forecasts the logarithm of the square root of monthly RV and not, as in this case, the level of daily RV. When constructing VaR and ES forecasts based on the volatility forecasts, we find significant differences in the performance of the HAR and the LSTM input models, where for these quantities, the HAR input models show better performance.

Fourth, the statistical analysis of the forecasting results pointed toward two hypotheses. First, the non-linear transformation through one LSTM cell is superior to all alternative transformations that we suggest, especially when only accounting for the most recent information in the transformed measure. Through a sequence of tests for superior predictive ability (SPA tests), we find that the non-linear transformation through one LSTM cell outperforms the alternatives. When only considering the most recent HF information, this result holds under conservative choices for the significance level. However, this result only holds for less conservative choices for the significance level (5%) for the setting where we account for dynamics in the transformed measure. So the non-linear transformation through one LSTM cell outperforms the MIDAS alternatives and the alternatives that allow for higher degrees of non-linearity by using a network of LSTM cells. This is very convenient since it circumvents the challenging task of finding the optimal network architecture for the transformation. Second, combining this transformed measure with the information on the past RV yields superior forecasting performance to all other models under consideration. Another sequence of SPA tests shows that the models that augment the information from the log RV with the most recent transformation from one LSTM cell significantly outperform all alternative models, including the benchmarks. When augmented by the most recent transformation from one LSTM cell, there are no significant differences between the model that uses an LSTM on past log RV and the model that uses the HAR. So also in this case, the HAR models' lagged daily, weekly, and monthly averages are approximating the long persistence in the volatility equally well as the LSTM cell.

Our analysis thus directs to a new type of HAR model that augments the classical HAR by a non-linear transformation of the HF returns within a day. These results are in line with the findings of Rahimikia and Poon (2020a), who also find that their proposed HAR model augmented by HF limited order book and news sentiment data shows superior forecasting performance. However, the information we utilize for the augmentation does not stem from an auxiliary source such as news feeds but from the same information used to construct the RV estimator. Our resulting models can outperform some of the most popular benchmark models in the literature, such as ARFIMA models, the HAR, the CHAR, and the HARQ model. A natural extension of the presented work would be to use Bi-Power Variation and Realized Quarticity measures as additional inputs for the forecasting task. One could then assess, whether in this case, there are also gains in the forecasting performance through augmenting this model with the non-linear transformation of the HF returns through one LSTM cell.

## Data Availability Statement

The raw data supporting the conclusions of this article will be made available by the authors, without undue reservation.

## Author Contributions

CM contributed to the conceptualization of the idea, implemented the code, and wrote the manuscript.

## Conflict of Interest

The author declares that the research was conducted in the absence of any commercial or financial relationships that could be construed as a potential conflict of interest.

## Publisher's Note

All claims expressed in this article are solely those of the authors and do not necessarily represent those of their affiliated organizations, or those of the publisher, the editors and the reviewers. Any product that may be evaluated in this article, or claim that may be made by its manufacturer, is not guaranteed or endorsed by the publisher.

## Footnotes

^1^The hyperbolic tangent function applied to value *x* is *tanh*(*x*) = [exp (*x*) − exp (−*x*)]/[exp (*x*) + exp (−*x*)]. It is a sigmoid function rescaled to the interval (−1, 1).

^2^The sigmoid function applied to value *x* is defined as σ(*x*) = 1/[1 − exp (−*x*)].

^3^There exists no strictly consistent loss function for the ES alone (Gneiting, 2011).

^4^The Binomial test tests whether positive and negative sign changes in the loss differential of two models are equally likely. It is also known as the Diebold Mariano Sign test.
