# Supplementary material for: Artificial Neural Network Based Non-linear Transformation of High-Frequency Returns for Volatility Forecasting
Source: Front Artif Intell. 2022 Feb 11;4:787534. doi: 10.3389/frai.2021.787534 (PMC8873984; doi:10.3389/frai.2021.787534)
Supplement: Supplementary file 1 [file Presentation_1.pdf]

SUPPLEMENTARY MATERIAL

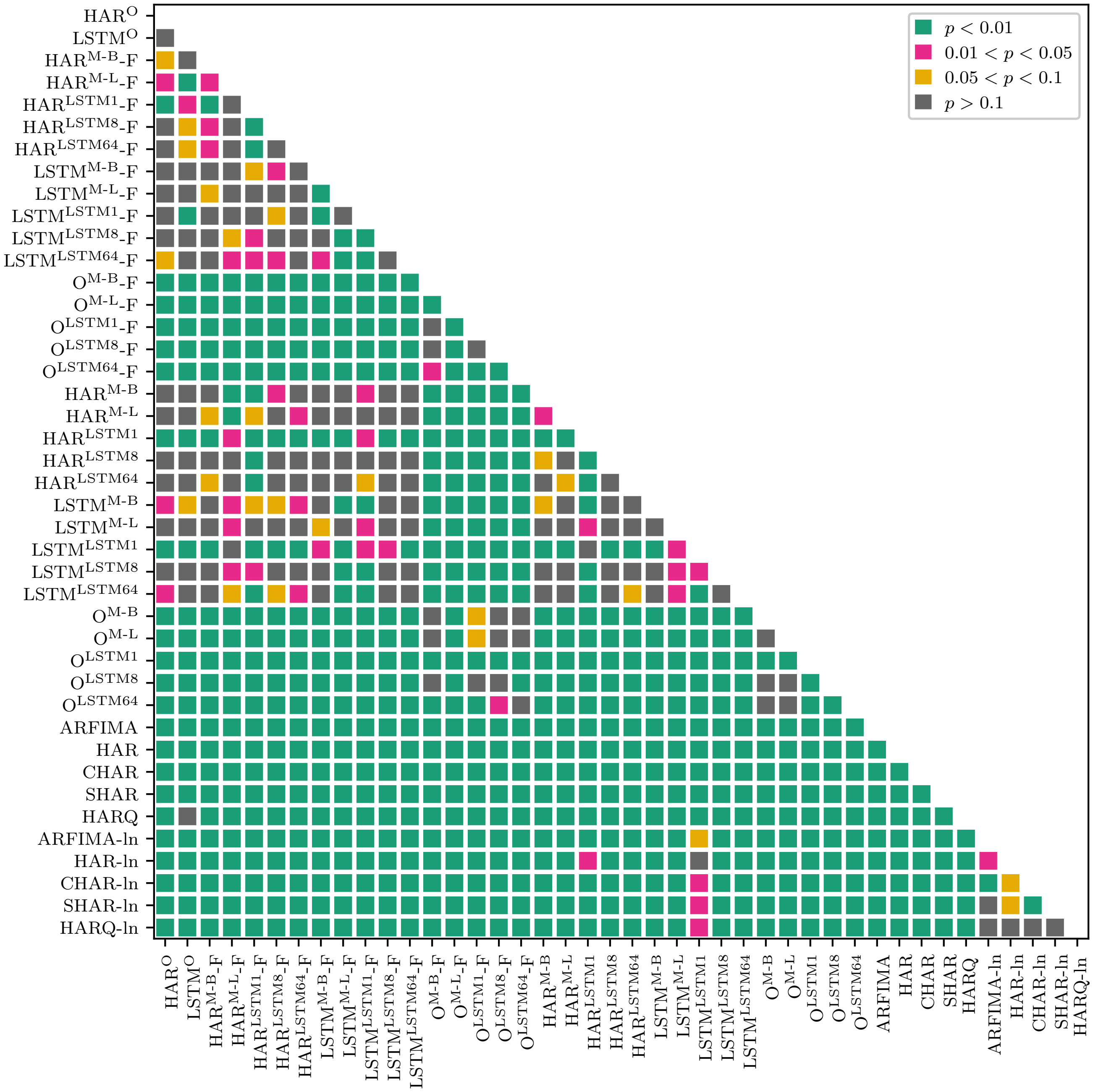

FIGURE 1 | p-values of a Binomial test for equal forecasting performance for the QLIKE loss depicted.

We test the models in the rows against the model specified by the column. Since the test result is symmetric, we report the lower triangle. A rejection of the H0 indicates significant differences in the forecasting performance of the two models.

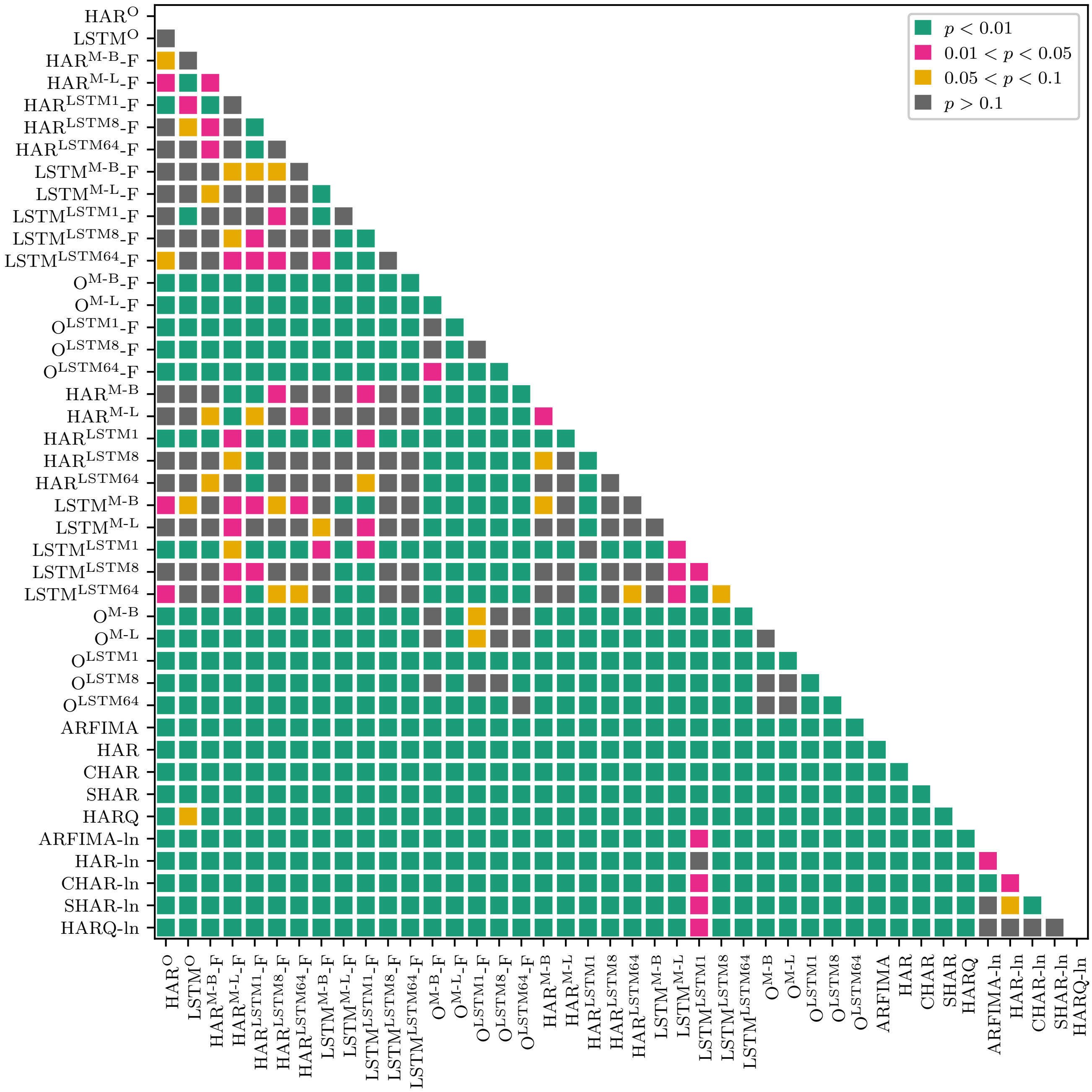

FIGURE 2 | p-values of a Binomial test for equal forecasting performance for the squared error loss depicted.

We test the models in the rows against the model specified by the column. Since the test result is symmetric, we report the lower triangle. A rejection of the H0 indicates significant differences in the forecasting performance of the two models.

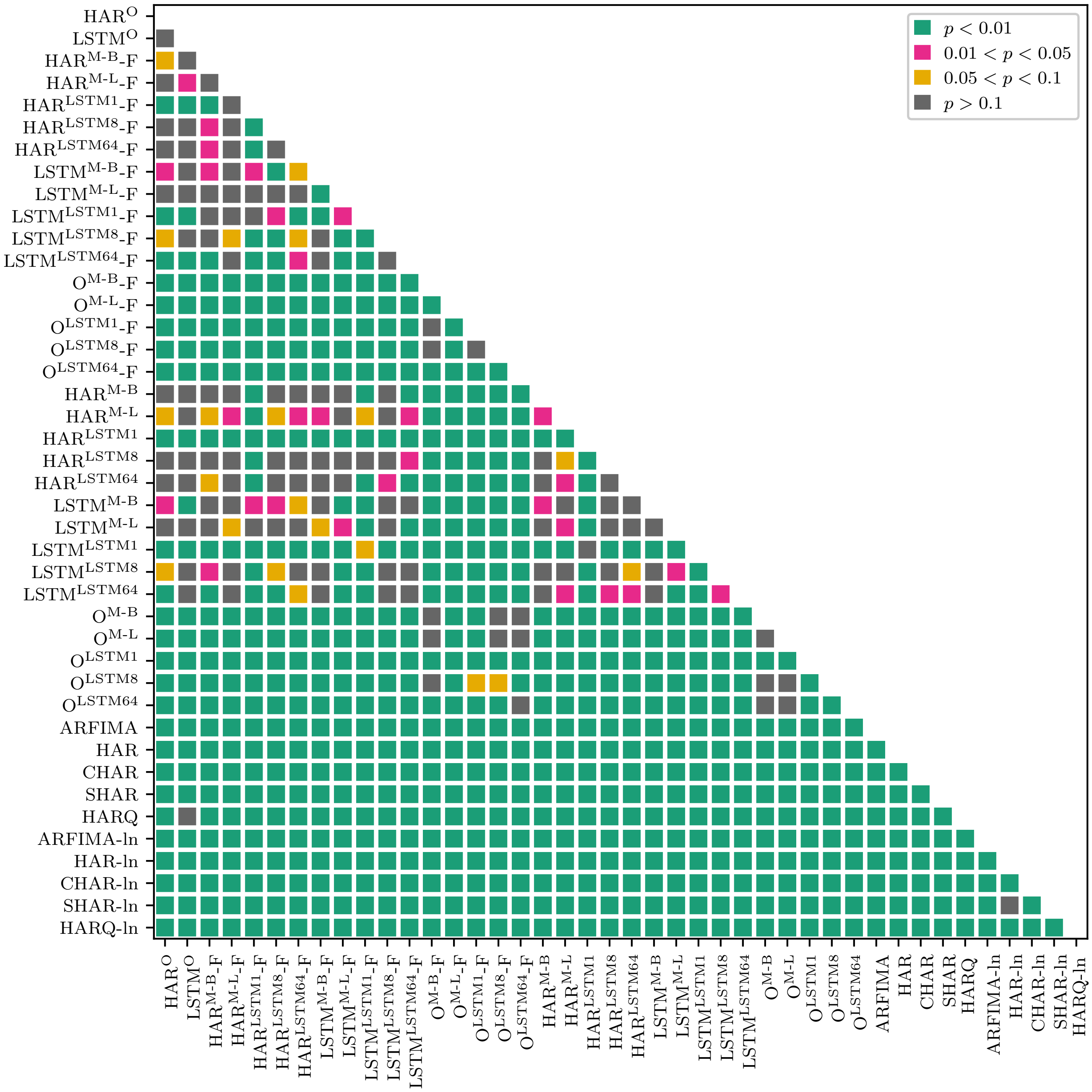

FIGURE 3 | p-values of a Binomial test for equal forecasting performance for the $VaR_{1\%}$ score loss depicted.

We test the models in the rows against the model specified by the column. Since the test result is symmetric, we report the lower triangle. A rejection of the H0 indicates significant differences in the forecasting performance of the two models.

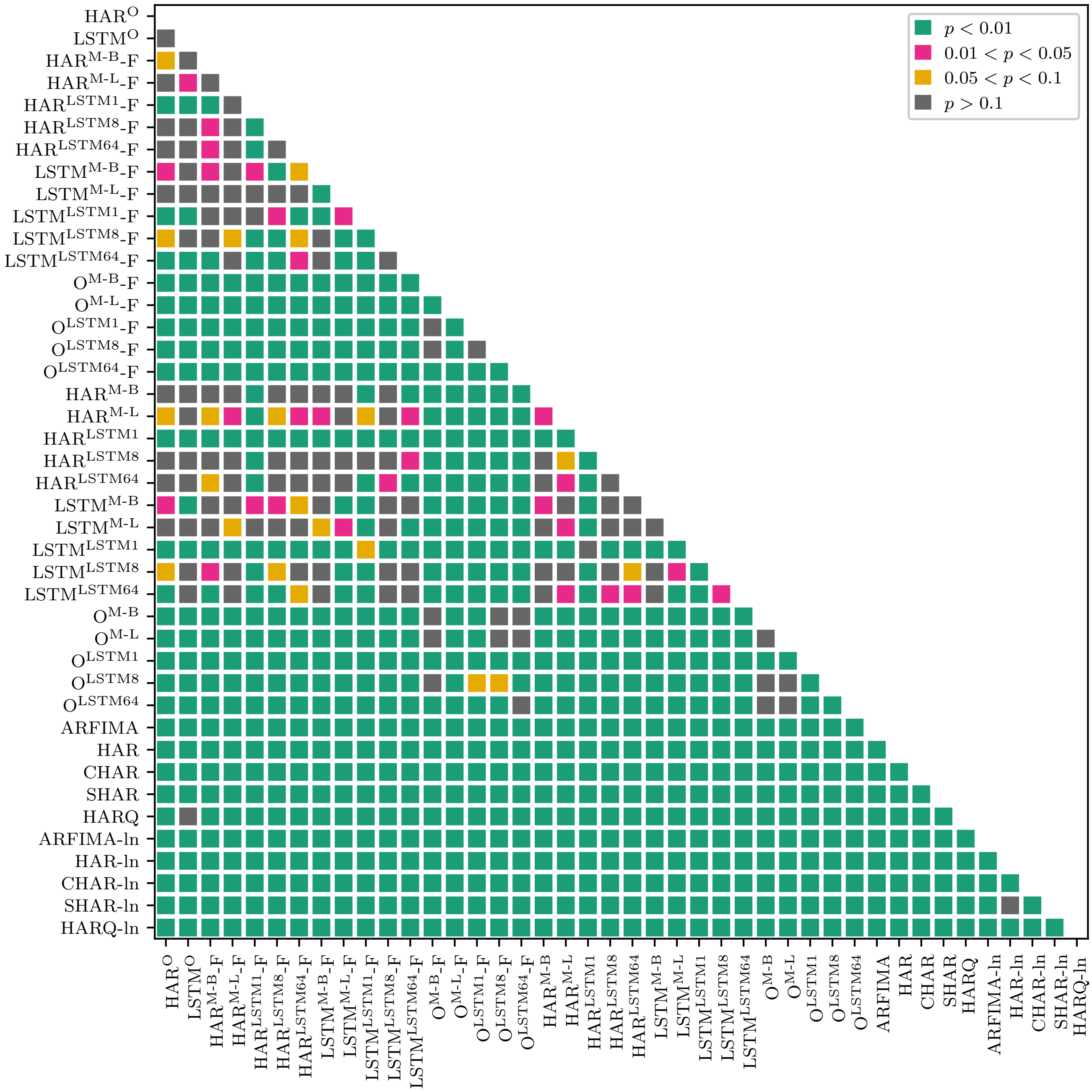

FIGURE 4 | p-values of a Binomial test for equal forecasting performance for the VaR $ES_{1\%}$ score loss depicted.

We test the models in the rows against the model specified by the column. Since the test result is symmetric, we report the lower triangle. A rejection of the H0 indicates significant differences in the forecasting performance of the two models.

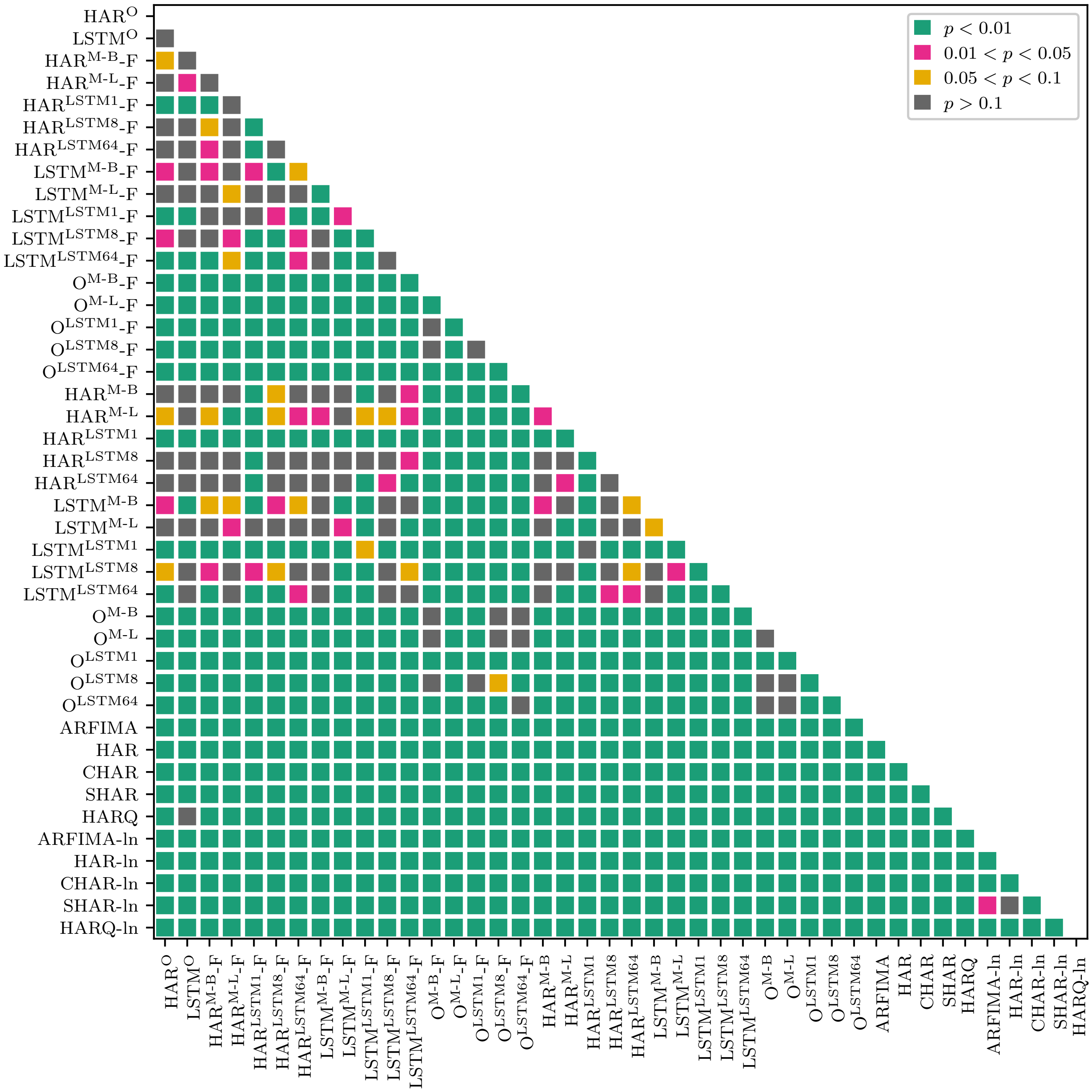

FIGURE 5 | p-values of a Binomial test for equal forecasting performance for the $VaR_{2.5\%}$ score loss depicted.

We test the models in the rows against the model specified by the column. Since the test result is symmetric, we report the lower triangle. A rejection of the H0 indicates significant differences in the forecasting performance of the two models.

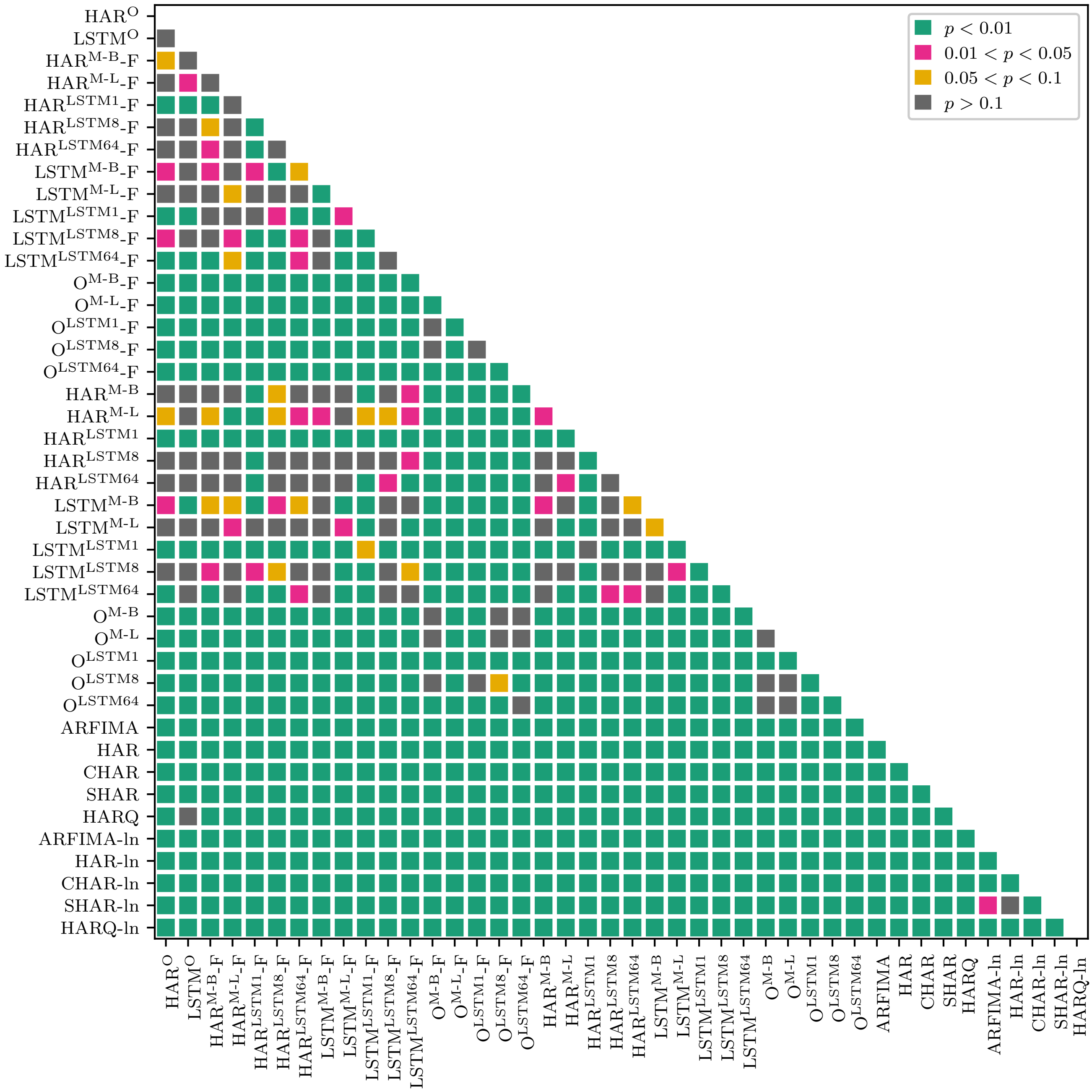

FIGURE 6 | p-values of a Binomial test for equal forecasting performance for the VaR $ES_{2.5\%}$ score loss depicted.

We test the models in the rows against the model specified by the column. Since the test result is symmetric, we report the lower triangle. A rejection of the H0 indicates significant differences in the forecasting performance of the two models.
